# A Precise Activity System for Pharmacogenetics Based on Estimation of *cyp2d6* allelic Percentage Activity from the International CEPAM Database

**DOI:** 10.1002/cpt.70560

**Published:** 2026-11-11

**Authors:** Fenja Klima, Thomas Helland, Robin Michelet, Anna M. Mc Laughlin, Jesse J. Swen, Henk‐Jan Guchelaar, Fabienne Thomas, Melanie White‐Koning, Ron H. N. van Schaik, Ron H. J. Mathijssen, Werner Schroth, Matthias Schwab, Gunnar Mellgren, Andrea Gaedigk, Daniel L. Hertz, Charlotte Kloft, the CEPAM consortium, Fenja Klima, Thomas Helland, Robin Michelet, Anna M. Mc Laughlin, Jesse J. Swen, Henk‐Jan Guchelaar, Fabienne Thomas, Melanie White‐Koning, Ron H.N. van Schaik, Ron H.J. Mathijssen, Werner Schroth, Matthias Schwab, Gunnar Mellgren, Andrea Gaedigk, Daniel L. Hertz, Charlotte Kloft, Stijn L.W. Koolen, Patrick Neven, Bram C. Agema, Florence Dalenc, Thomas Mürdter, Hiltrud Brauch, Anabel Sanchez‐Spitman, Thais Almeida, Jeanine Nardin, José Claudio Casali‐da‐Rocha, Rosa Esther Moo‐Puc, Jorge Aarón Rangel‐Mendez, Gwendolyn McMillin, Ewa E. Hennig, Elżbieta Brewczyńska, Marina Venzon Antunes, Vincent Haufroid, Linda Thorén, Lisa Madlensky, John Pierce, Yusuke Nakaumra, Michiaki Kubo, Hitoshi Zembutsu, João Paulo Bianchi Ximenez, Vera Lucia Lanchote, James M. Rae, Daniel F Hayes, Vered Stearns, Todd C. Skaar, Zeruesenay Desta, Stuart A. Scott, Robert J. Desnick, In Hae Park, Hye In Woo, Soo‐Youn Lee, Ana Fernandez‐Santander, Alicia Romero‐Lorca, Apolonia Novillo Villajos, María Gaibar Alonso, Harriet Johansson, Bernardo Bonanni, Andrea DeCensi, Howard Gurney, Rosemary Balleine, William J. Irvin, Howard L. McLeod, Matthew P. Goetz, Joel M. Reid, Vera J. Suman, Nutthada Areepium, Wanaporn Charoenchokthavee, Diana Eccles, William Tapper, Balram Chowbay, Chiea Chuen Khor, Elaine Lim Hsuen, Nathalie K. Zgheib, Cécile Arellano

**Affiliations:** ^1^ Department of Clinical Pharmacy and Biochemistry Institute of Pharmacy, Freie Universität Berlin Berlin Germany; ^2^ Graduate Research Training Program PharMetrX Berlin/Potsdam Germany; ^3^ Department of Clinical Pharmacy University of Michigan College of Pharmacy Ann Arbor Michigan USA; ^4^ Hormone Laboratory, Department of Medical Biochemistry and Pharmacology Haukeland University Hospital Bergen Norway; ^5^ Department of Clinical Science University of Bergen Bergen Norway; ^6^ Pharmetheus AB Uppsala Sweden; ^7^ Department of Clinical Pharmacy & Toxicology Leiden University Medical Center Leiden The Netherlands; ^8^ Inserm, CRCT Institut Claudius Regaud, IUCT‐Oncopole, Univ Toulouse Toulouse France; ^9^ Inserm, CRCT, Univ Toulouse Toulouse France; ^10^ Department of Clinical Chemistry Erasmus University Medical Center Rotterdam The Netherlands; ^11^ Department of Medical Oncology Erasmus MC Cancer Institute, Erasmus University Medical Center Rotterdam The Netherlands; ^12^ Dr. Margarete Fischer‐Bosch Institute of Clinical Pharmacology Stuttgart Germany; ^13^ University Tübingen Tübingen Germany; ^14^ Cluster of Excellence iFIT (EXC 2180) “Image‐Guided and Functionally Instructed Tumor Therapies” University of Tübingen Tübingen Germany; ^15^ German Cancer Consortium (DKTK) and German Cancer Research Center (DKFZ), Partner Site Tübingen Tübingen Germany; ^16^ Departments of Clinical Pharmacology, Biochemistry and Pharmacy University of Tübingen Tübingen Germany; ^17^ Division of Clinical Pharmacology, Toxicology & Therapeutic Innovation Children's Mercy Research Institute (CMRI) Kansas City Missouri USA; ^18^ University of Missouri‐Kansas City School of Medicine Kansas City Missouri USA

## Abstract

The polymorphic enzyme CYP2D6 contributes to the metabolism of many frequently prescribed drugs, causing substantial variability in drug exposure and treatment outcomes. Genotype‐based dosing recommendations currently rely on drug‐agnostic categorical *CYP2D6* allele activity values (AVs), potentially leading to imprecise CYP2D6 phenotype classification and under‐/overdosing of numerous medications. To further advance precision dosing, we developed a *CYP2D6* percentage activity (*CYP2D6*‐PA) framework by estimating *CYP2D6* allelic percentage activity (*CYP2D6*‐APA) from 0% (no activity) to 100% (CYP2D6*1‐normalized reference activity) using tamoxifen and its active metabolite Z‐endoxifen as a model substrate. By leveraging nonlinear mixed‐effects modeling of the international CEPAM database (>7,000 patients from 31 studies), bias‐corrected *CYP2D6*‐APAs were estimated, considering global allele distributions, additional covariates, and limited *CYP2D6* genotyping. Estimated *CYP2D6*‐APAs spanned 0%–100%, several deviating substantially from their categorical AVs. In particular, CYP2D6*2 and *35 (normal function, AV = 1), and *17 and *29 (decreased function, AV = 0.5) showed lower *CYP2D6*‐APAs of 64–68% and 29%, respectively. Conversely, CYP2D6*9 (decreased function, AV = 0.25) showed a higher *CYP2D6*‐APA (52%). Each additional allele copy exhibited 68% of the activity of the first copy, less than the twofold increase assumed by AVs. Neglecting limited genotyping (alleles misclassified as reference CYP2D6*1), caused *CYP2D6*‐APA overestimation by 1.1‐fold. Using *CYP2D6*‐APAs instead of AVs translated to changes in predicted individual Z‐endoxifen concentrations (−22.3% to 40.0%). While substrate specificity of empirically derived robust tamoxifen *CYP2D6*‐APAs requires evaluation, *CYP2D6*‐PA provides a framework applicable to other substrates/pharmacogenes. Thus, *CYP2D6*‐PA lays the foundation for revising genotype‐to‐phenotype translation systems, improving precision of pharmacogenetics‐guided dosing and optimizing treatment outcomes upon prospective validation.

**Study Highlights**

**WHAT IS THE CURRENT KNOWLEDGE ON THE TOPIC?**

*CYP2D6* genotype plays an important role in the metabolism of many frequently used drugs. However, the translation from *CYP2D6* genotype to CYP2D6 phenotype relies on categorical activity values (AV) and may not precisely reflect allele activity

**WHAT QUESTION DID THIS STUDY ADDRESS?**

*CYP2D6* allele percentage activities (*CYP2D6*‐APAs) were estimated using tamoxifen as a model substrate leveraging a multi‐study database with >7,000 patients using nonlinear mixed‐effects modeling and considering additional impacting factors and incomplete genotype data.

**WHAT DOES THIS STUDY ADD TO OUR KNOWLEDGE?**

Some estimated *CYP2D6*‐APAs deviated substantially from their categorical CPIC‐assigned AVs. For example, CYP2D6*2 and *35 (normal function, AV = 1) as well as *17 and *29 (decreased function, AV = 0.5) showed lower *CYP2D6*‐APAs than their current assignment while CYP2D6*9 (decreased function, AV = 0.25) showed a higher *CYP2D6*‐APA. This highlighted the potential of a more precise system to translate *CYP2D6* genotype to CYP2D6 phenotype for tamoxifen and other CYP2D6 substrates.

**HOW MIGHT THIS CHANGE CLINICAL PHARMACOLOGY OR TRANSLATIONAL SCIENCE?**

*CYP2D6* percentage activity (*CYP2D6‐*PA) represents a more precise and intuitive system to translate *CYP2D6* genotype to CYP2D6 phenotype. The developed framework can be applied to derive substrate‐specific *CYP2D6*‐APAs for other CYP2D6 substrates or percentage activities for other pharmacogenes, which may ultimately contribute to more precise personalized treatment and facilitate uptake of pharmacogenetics‐guided dosing upon prospective validation in clinical practice.

Cytochrome P450 2D6 (CYP2D6) is the major metabolic pathway for ≥20% of the most commonly prescribed drugs,^1^ including metabolic elimination of beta‐blockers^2^ (e.g., metoprolol and carvedilol) used in hypertension, heart failure, and post‐myocardial infarction as well as antidepressant medication classes^3^, ^4^ (e.g., tricyclics and serotonin reuptake inhibitors). Additionally, CYP2D6 is the primary enzyme responsible for metabolic activation of commonly used prodrugs, including analgesics^5^ (e.g., codeine/hydrocodone and tramadol) and the anticancer drug tamoxifen (TAM).^6^ Genetic variations in the highly polymorphic *CYP2D6* gene lead to tremendous variability in patients' CYP2D6 enzymatic activity (i.e., phenotype),^7^ affecting plasma concentrations of a drug's active molecular moiety (i.e., pharmacokinetics (PK)) and treatment outcomes. The Clinical Pharmacogenetics Implementation Consortium (CPIC) has published seven evidence‐based guidelines for personalizing treatment of these and other drug classes^2^, ^3^, ^4^, ^5^, ^6^, ^8^, ^9^ widely used in clinical practice to inform personalized treatment decisions in patients with known *CYP2D6* genotype.^10^

While the crucial role of *CYP2D6* genotype in treatment optimization is well established, translation of an individual's *CYP2D6* genotype to their CYP2D6 phenotype remains challenging. Currently, a consensus process from CPIC and the Dutch Pharmacogenetics Working Group (DPWG) translates genotypes into a categorical activity score (AS)^11^: Each *CYP2D6* haplotype, or star (*) allele,^12^ is assigned a categorical activity value (AV) of 0 (no function), 0.25/0.5 (decreased function), 1 (normal function), or integers >1 (increased function due to copy number variation (CNV)). AVs of a patient's two star alleles are summed to determine the patient's AS, which is translated to categorical phenotypes: poor (PM), intermediate (IM), normal (NM) or ultrarapid (UM) metabolizer used for treatment recommendations. However, imprecision in these categorical AV assignments may lead to imprecise estimation of a patient's CYP2D6 metabolic activity, which may cause phenotype misclassification and potential under‐/overdosing of various medications.

To further advance precision dosing, categorical AVs can be replaced with continuous *CYP2D6* allelic percentage activity (*CYP2D6*‐APA) precisely estimating each allele's relative metabolic activity from 0% (no activity) through 100% (CYP2D6*1‐normalized reference activity), or higher when considering gene duplications and increased function alleles. By averaging a patient's two *CYP2D6*‐APAs, an individual's CYP2D6 percentage activity (*CYP2D6*‐PA) could then be obtained on an intuitive scale (e.g., 100% = normal activity) as outlined in the CPIC/DPWG CYP2D6 genotype‐to‐phenotype consensus recommendation.^11^ Multiple analyses investigated *CYP2D6‐*PA for some alleles.^13^, ^14^, ^15^ However, a comprehensive assessment of *CYP2D6*‐APA covering a broad range of alleles and accounting for diverse populations, the foundation for establishing a *CYP2D6‐*PA system, is still lacking. To ensure coverage of common alleles across populations,^16^ a large dataset with patients from different biogeographical groups is warranted.^17^ Moreover, *CYP2D6*‐APA estimation requires consideration of other factors impacting the metabolic pathway of interest, including other CYP enzymes or patient characteristics.^18^, ^19^, ^20^, ^21^ Lastly, genotyping assays can vary considerably regarding alleles tested and limited genotyping may bias *CYP2D6*‐APA estimates.^16^, ^22^, ^23^ Nonlinear mixed‐effects (NLME) modeling can account for and quantify these factors when understanding PK and its variability between individuals, making it ideal to estimate *CYP2D6‐*APA in a large, diverse patient cohort with PK data from a CYP2D6‐metabolized drug.^24^

The international CYP2D6 Endoxifen Percentage Activity Model (CEPAM) database is one of the few datasets providing the unique combination of comprehensive data availability as well as ongoing clinical relevance of the drug. CEPAM pooled >30 clinical studies of patients receiving TAM treatment with PK data of TAM and its main active metabolite Z‐endoxifen (Z‐ENDX), *CYP2D6* genotype, and other relevant patient and treatment data.^25^ The prodrug TAM, a selective estrogen receptor modulator, is a cornerstone for hormone receptor‐positive breast cancer treatment.^26^ Z‐ENDX is formed via intermediate metabolites N‐desmethyl‐tamoxifen (main pathway) or 4‐hydroxytamoxifen. Whereas most metabolic steps are catalyzed by various CYP enzymes, the hydroxylation from N‐desmethyl‐tamoxifen to Z‐ENDX depends solely on CYP2D6.^18^

This work aimed to estimate *CYP2D6‐*APA for the most commonly observed *CYP2D6* alleles across diverse populations via NLME modeling of the CEPAM database using TAM as model substrate.^25^ It thereby aimed to lay a foundation for a refined system translating *CYP2D6* genotype to *CYP2D6*‐PA to inform precision dosing, ultimately optimizing therapeutic outcomes for the many CYP2D6 substrates used in clinical care.

## METHODS

### Clinical study database

CEPAM^25^ is a global database comprising clinical study cohorts of breast cancer patients treated with TAM and with *CYP2D6* genotype, and TAM and ENDX systemic concentrations. A previous version of the database was characterized elsewhere.^25^ All included studies were conducted in accordance with the Declaration of Helsinki and approved by respective institutional review boards. For each patient, at least one blood sample was collected and TAM and ENDX concentrations were quantified using various bioanalytical methods (**Table** **S1**). Some assays could not bioanalytically separate Z‐ENDX from the less active metabolite Z4′‐ENDX,^27^ measuring total ENDX (Z4′‐ENDX + Z‐ENDX) instead of Z‐ENDX as described previously.^25^

The data cleaning and imputation workflow has previously been published.^25^ Briefly, patients or studies were assessed for (1) eligibility for analysis, (2) distribution of TAM and ENDX measurements and (3) plausibility of reported dosing or PK; missing data were imputed depending on the magnitude of missingness and expected correlations (**Figure** **1**, **Supplementary** **Text S1**).

**Figure 1:**
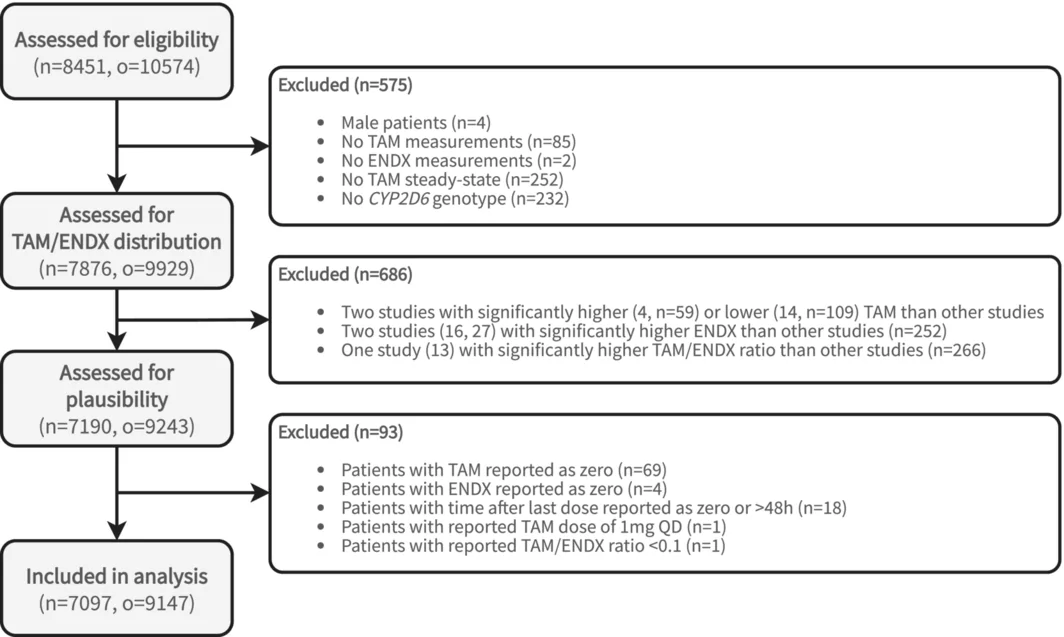
Flow diagram of patient and data exclusion. Data exclusion steps from the raw CYP2D6 Endoxifen Percentage Activity Model (CEPAM) database to the CEPAM analysis dataset illustrating number of patients (*n*) and number of pharmacokinetic samples (o) at each step. ENDX, endoxifen; QD, once daily; TAM, tamoxifen.

### Development of *CYP2D6* allelic percentage activity ( *CYP2D6* ‐APA)

The development of *CYP2D6*‐APA was based on a published PK NLME model built on a previous version of the CEPAM database.^25^ Briefly, the model consisted of one compartment for each of TAM and Z‐ENDX. Total ENDX was included using an empirical conversion factor between Z‐ENDX and total ENDX. To allow for a mechanistically interpretable implementation of *CYP2D6*‐APA, a stepwise workflow was adopted:

(1) Extension of PK NLME model.
(2) Modeling *CYP2D6*‐APA.
(3) Modeling CYP2D6 co‐medication.

#### Extension of PK NLME model

The structural, covariate, and residual variability (RUV) model were refined (**Supplementary** **Text S1**): To better reflect TAM metabolism, the empirical conversion factor calculating total ENDX was removed and the structural model extended by a semi‐mechanistic implementation of total ENDX, enabling the assessment of Z‐ENDX formation independent from Z4′‐ENDX. Additionally, Z‐ENDX formation was split into a CYP2D6‐dependent and CYP2D6‐independent pathway, allowing *CYP2D6*‐APA to reach 0% later on. Next, covariates were removed and re‐investigated during covariate analysis if they were affected by structural changes or new data supported re‐implementation. Finally, to account for different levels of missing data between studies with one vs. multiple sampling timepoints, two separate RUV models per analyte were tested.

#### Modeling *CYP2D6* ‐APA

*CYP2D6*‐APA was estimated on a percentage scale defined by two reference points: (1) The CYP2D6*5 gene deletion was assigned an activity of 0% (no function) and (2) CYP2D6*1 100% (reference function).^12^ To quantify the impact of CNVs, the relative activity increase of additional allele copies was estimated by a scaling factor (*SF*) constrained between 0 and 1:

(1) $${\mathrm{APA}}_{\mathrm{CNV}}={\mathrm{APA}}_{\text{Copy}1}+{\mathrm{APA}}_{\text{Copy}1}*\text{NxCNV}*\mathrm{SF},$$

with *APA*_*Copy1*_ and *APA*_*CNV*_ referring to the *CYP2D6‐*APA of the first and all allele copies and *NxCNV* as the number of additional copies. To obtain a patient's *CYP2D6‐*PA representing their diplotype activity, their two *CYP2D6‐*APAs were averaged, assuming no interaction between alleles. To challenge this assumption, allele interaction was investigated (**Supplementary** **Text S1**). *CYP2D6‐*PA (*PA*) was then implemented as relative change of typical, that is, reference, CYP2D6‐dependent Z‐ENDX formation (*CL*_*CYP2D6,reference*_ for 100% *CYP2D6‐*PA):

(2) $$\begin{array}{c} {\mathrm{CL}}_{\mathrm{CYP}2D6}={\mathrm{CL}}_{\mathrm{CYP}2D6,\text{reference}}*\mathrm{PA} \end{array}$$

Alleles were included stepwise following their frequency in CEPAM: common (≥1%, *n* ≥ 142 occurrences), rare (≥0.1%, *n* ≥ 14) and very rare (<0.1%, *n* < 14) alleles. For each allele, estimation of its activity was attempted. Estimates were fixed to zero if they were close to zero with high relative standard error (RSE) or assigned the categorical AV obtained from ClinPGx^12^ if unidentifiable due to insufficient allele frequency (<0.1%).

#### Modeling CYP2D6 co‐medication

To quantify the impact of co‐medication on CYP2D6 activity, weak/moderate and strong CYP2D6 inhibitor effects were included as categorical covariates implemented as relative change on *CL*_*CYP2D6,reference*_.

### Analysis of CYP2D6‐independent influential factors

After implementing *CYP2D6*‐APA, CYP2D6‐independent covariates were investigated. Covariates were pre‐selected if their impact was plausible, a trend was observed in plots of covariate vs. individual deviations from population parameter or covariates had been removed during model extension. Statistical significance of covariate effects was tested using stepwise covariate modeling^28^ with significance levels of α = 0.05 for forward inclusion and α = 0.001 for backward deletion as previously described.^25^ **Supplementary** **Text S1** details investigated covariate relationships and a sensitivity analysis assessing covariate imputation.

### Assessing bias in *CYP2D6* ‐APA through limited genotyping

Using the otherwise final model, the impact of limited *CYP2D6* genotyping on estimated *CYP2D6*‐APA was quantified. Roche AmpliChip represented the most comprehensive and commonly used assay in the dataset (*n* = 2,774 patients, **Table** **S2**) and was used as reference for complete genotype data. To account for limited testing of other assays, resulting in CYP2D6*1 default allele assignments, assay‐specific deviations of apparent CYP2D6*1 reference activity from 100% were assessed: First, reference allele frequency was obtained from data genotyped with Roche AmpliChip and stratified by biogeographical group if a group contained sufficient individuals (*n* ≥ 100). Second, for each genotyping assay, alleles included were compared with Roche AmpliChip and missing alleles identified (**Table** **S2**). These alleles were assumed to be misclassified as CYP2D6*1 in the respective assay and to impact apparent reference activity as follows:

(3) $$\begin{array}{c} {\mathrm{APA}}_{*1,\text{apparent}}=\frac{{\mathrm{APA}}_{*1}*{\text{freq}}_{*1}+\sum_{i=1}^{i=n}A{\mathrm{PA}}_{\text{misclassfied},i}*{\text{freq}}_{\text{misclassified},i}\;}{{\text{freq}}_{*1}+\sum_{i=1}^{i=n}{\text{freq}}_{\text{misclassified},i}}, \end{array}$$

corresponding to a weighted average of *CYP2D6*‐APAs lumped into the *CYP2D*6*1 reference, where *freq* is the reference allele frequency of the corresponding allele. Missing identification of CNVs was considered for CYP2D6*1xN, *2xN, *4xN and *41xN, the most commonly observed gene duplications^16^ (**Supplementary** **Text S1**). Third, apparent CYP2D6*1 reference activity (APA_*1,apparent_) was implemented in the model, replacing the fixed reference activity of 100%, allowing to simultaneously estimate apparent reference activity and *CYP2D6*‐APA. Finally, using the final *CYP2D6*‐APAs, individual *CYP2D6*‐PAs of CEPAM patients were obtained and compared with their CPIC‐assigned CYP2D6 AS.

### Assessment of impact of clinical influential factors on Z‐ENDX

Covariate effects in the final model were assessed via explained interindividual variability and theoretical covariate effect on minimal Z‐ENDX concentration using a forest plot (**Supplementary** **Text S1**). Additionally, using either the *CYP2D6* AS or *CYP2D6*‐PA, real‐world population and individual steady‐state minimal Z‐ENDX concentrations at 24 hours after the last dose at a standard tamoxifen dose of 20 mg/day were simulated for each patient in the CEPAM dataset assuming treatment adherence and reported baseline patient covariates.

### Model performance and software

Models were evaluated with respect to parameter plausibility and precision, goodness‐of‐fit plots, and suitable numerical metrics. Detailed information on assessing model performance and software used is summarized in **Supplementary** **Text S1**.

## RESULTS

### Clinical study database

The raw CEPAM database comprised 8,451 patients with 10,574 PK sampling timepoints from 36 clinical studies. A total of 16.0% of patients were excluded for the following reasons (see **Figure** **1**, **Supplementary** **Text S2** for details): 6.80% (*n* = 575) due to non‐eligibility for analysis; 8.12% (5 studies, *n* = 686) due to deviating TAM or ENDX concentrations without explanatory factors; 1.10% (*n* = 93) due to implausible reported dosing, sampling times, or TAM or ENDX measurements.

The analysis dataset comprised 7,097 patients with 9,147 PK sampling timepoints from 31 clinical studies. Patient characteristics, information on dosing, and PK sampling are summarized in **Table** **1** and **Figure** **S1**. Z‐ENDX and total ENDX were measured in 86.5% and 13.5% of patients, respectively. For 93.7% of patients from 27 studies, only one sampling timepoint was available, while 6.31% of patients from six studies had multiple (median: 4).

**Table 1:** Clinical study and population characteristics in the CYP2D6 Endoxifen Percentage Activity Model (CEPAM) analysis dataset without *CYP2D6* genotypes (**Table 2**)

Characteristic	Number of patients or PK samples (%) or median (range)
Number of evaluable patients	7,097
Number of studies	31
**Dosing or PK sampling**	
Number of evaluable PK samples (after re‐classification)	
Tamoxifen	9,147 (100)
Z‐endoxifen	6,136 (86.5)
Z‐4′‐endoxifen	1,375 (19.4)
Total endoxifen	961 (13.5)
Patients with one or multiple PK samples	
One PK measurements	6,649 (93.7)
Multiple PK measurements	448 (6.31)
Tamoxifen dose (per PK sample)	
5 mg QD	154 (1.68)
10 mg QD	44 (0.481)
15 mg QD	2 (0.0219)
20 mg QD	8,567 (93.7)
30 mg QD	76 (0.831)
40 mg QD	304 (3.32)
Missing data^a^	0 (0)
Time after last dose (h) (per PK sample)	
Median [range]	15.1 [0.0167–48.0]
Missing data^a^	5,438 (59.5)
**CYP2D6‐related**	
CYP2D6 inhibitor co‐medication^b^	
None	3,817 (53.8)
Weak	206 (2.90)
Moderate	7 (0.0986)
Strong	64 (0.902)
Missing data^a^	3,003 (42.3)
**CYP2D6‐independent**	
Age at baseline (years)	
Median	51 [22–95]
Missing data^a^	466 (6.57)
Body weight at baseline (kg)	
Median	68 [32–190]
Missing data^a^	2050 (28.9)
Biogeographical group	
White	3,434 (48.4)
Asian	993 (14.0)
Black/African American	451 (6.35)
Other	68 (0.958)
Missing data^a^	2,151 (30.3)
*CYP3A4* genotype‐predicted phenotypes	
Poor metabolizer	3 (0.0423)
Intermediate metabolizer	230 (3.24)
Normal metabolizer	2,487 (35.0)
Missing data^a^	4,377 (61.7)
CYP3A4 inducer co‐medication^b^	
None	2,233 (31.5)
Weak	23 (0.324)
Moderate	0 (0)
Strong	5 (0.0705)
Missing data^a^	4,836 (68.1)
*CYP2C9* genotype‐predicted phenotypes	
Poor metabolizer	28 (0.395)
Intermediate metabolizer	441 (6.21)
Normal metabolizer	1,099 (15.5)
Missing data^a^	5,529 (77.9)
Adherence (as reported in the database, per PK sample)	
Yes	3,178 (34.7)
No	175 (1.91)
Missing data^a^	5,794 (63.3)

PK, pharmacokinetic(s); QD, once daily.

^a^ Missing data were imputed for analysis as described in **Supplementary Text S1**.

^b^ Inhibitor/inducer strength as defined by Flockhart Table available at: https://medicine.iu.edu/internal‐medicine/specialties/clinical‐pharmacology/drug‐interaction‐flockhart‐table.

### Development of *CYP2D6* ‐APA

**Figure** **2** **a** illustrates the final model after extension, integration of *CYP2D6*‐APA and subsequent covariate analysis (NONMEM code on GitHub: https://github.com/Kloft‐Lab/Klima‐et‐al‐2026‐model‐code). All model parameters and *CYP2D6*‐APAs were estimated with good precision (≤30% RSE, **Tables** **2** **and** **3**). TAM and Z‐ENDX concentrations were described well with a slight underprediction of high concentrations and comparable performance was observed for both total ENDX and Z‐ENDX (**Figure** **2** **b,c**, **Figure** **S2**).

**Figure 2:**
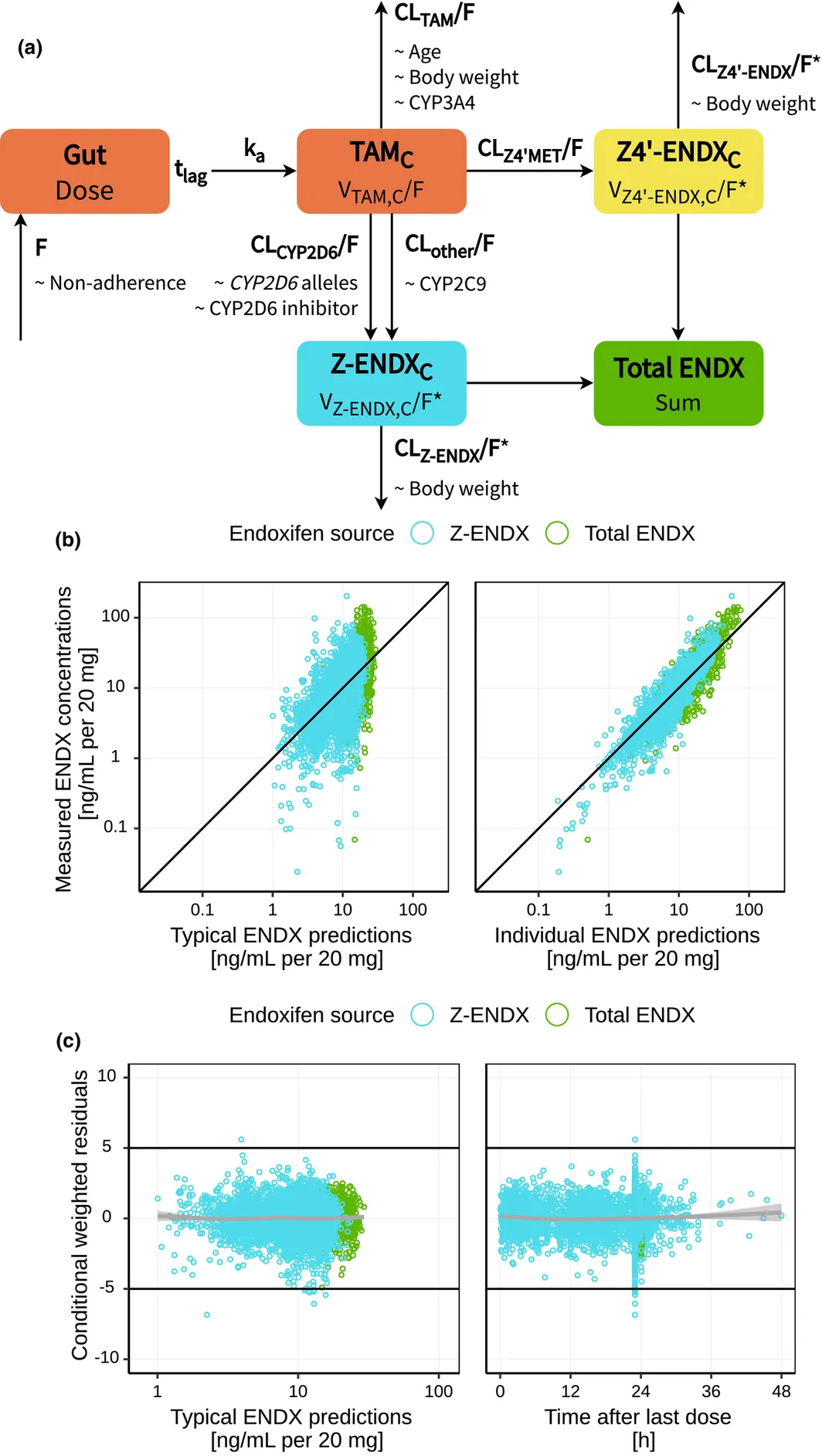
Schematic representation and goodness‐of‐fit (GOF) plots for extended model of tamoxifen (TAM) and endoxifen (ENDX). (**a**) Tamoxifen (TAM) was orally administered (bioavailability F) and absorbed via a first‐order process (k_a_) with lag time (t_lag_) to the central compartment with apparent volume of distribution (V) V_TAM,C_/F. TAM could be metabolized to Z‐ENDX with apparent formation CL_CYP2D6/other_/F, to Z4′‐ENDX with CL_Z4′MET_/F or to other metabolites with apparent clearance (CL) CL_TAM_/F. Z‐ENDX was eliminated from its central compartment V_Z‐ENDX,C_/F with CL_Z‐ENDX_/F. Z4′‐ENDX eliminated from its central compartment V_Z4′‐ENDX,C_/F with clearance CL_Z4′‐ENDX_/F. Total ENDX was modeled as sum of Z‐ENDX and Z4′‐ENDX. All formation and elimination pathways were modeled as first‐order processes. Covariate‐parameter relationships are depicted with tildes (~). Parameters marked with a star (*) were fixed. (**b**) GOF plots for the final extended model comparing population (left) and individual (right) ENDX predictions with the measured concentrations, colored by ENDX type. Predictions and concentrations are dose‐normalized to 20 mg TAM dosing. The diagonal black line marks the line of identity. **(c)** Diagnostic plots of conditional weighted residuals (CWRES) for ENDX predictions vs. population ENDX predictions (left) and time after last dose (right) using the final extended model, colored by ENDX type. The black horizontal lines mark CWRES of +5 and −5.

**Table 2:** Overview of *CYP2D6* alleles and their frequencies in the CYP2D6 Endoxifen Percentage Activity Model (CEPAM) analysis dataset with Clinical Pharmacogenetics Implementation Consortium (CPIC)‐assigned categorical activity values, estimated allelic percentage activity, and relative bias through limited genotyping. Alleles are ordered by categorical activity value and within each category by star alleles

Categorical activity value^a^	*CYP2D6* allele	Allele frequency (*n* = 14,194 alleles)		Estimated allelic percentage activity [95% CI], %	
		Absolute	Relative, %	Considering limited genotyping	Not considering limited genotyping (relative bias, %)
1 (normal function)	*1 b	6,296	44.4	100	100
	*2 c	1,906	13.4	64.2 [60.4–68.3]	69.8 [65.9–74.6] (8.85)
	*34 d	4	0.0282	100	100
	*35	257	1.81	68.3 [57.3–79.5]	76.2 [64.2–88.8] (11.5)
	*39	20	0.141	70.3 [34.4–93.5]	75.0 [44.3–95.6] (6.66)
0.5 (decreased function)	*14 d	5	0.0352	50	50
	*17 c	170	1.20	29.4 [20.0–38.6]	31.4 [21.9–42.4] (7.01)
	*29 c	69	0.486	29.1 [13.5–44.1]	31.4 [14.6–48.8] (7.62)
0.25 (decreased function)	*9 c	313	2.21	52.1 [44.2–60.9]	59.6 [50.0–68.8] (14.3)
	*10 c	1,038	7.31	23.2 [18.8–28.2]	25.2 [20.3–30.1] (8.39)
	*41 c	1,076	7.58	16.2 [13.0–19.4]	18.0 [14.4–21.6] (10.5)
0 (nonfunctional)	*3 c, e	143	1.01	0	0
	*4 c, e	2,171	15.3	0	0
	*5 c, e	543	3.83	0	0
	*6 c, e	119	0.838	0	0
	*7 f	48	0.338	16.2 [8.10–24.6]	17.1 [3.40–36.2] (5.43)
	*11 d	1	0.00704	0	0
	*12 d	2	0.0141	0	0
	*13 d	3	0.0211	0	0
	*15 d	2	0.0141	0	0
	*19 d	1	0.00704	0	0
	*21 d	2	0.0141	0	0
	*31 d	1	0.00704	0	0
	*36 d	1	0.00704	0	0
	*40 d	2	0.0141	0	0
	*68 d	1	0.00704	0	0
xN (increased)	CNVs^c^, ^g^	291	2.05	68.4 [49.4–89.9]	67.1 [47.4–89.3]

Allele frequencies stratified by biogeographical group obtained from patients genotyped with Roche AmpliChip are summarized in **Table** **S3**.

CI, confidence interval; CNV, copy number variation (xN indicates two or more copies).

^a^ Categorical activity values were obtained from the ClinPGx database.^12^

^b^ Values were fixed as reference points for percentage activity.

^c^ Tier 1 alleles recommended for clinical genetic testing.^16^

^d^ Values were fixed to categorical activity value due to low allele frequency.

^e^ Values were fixed to 0 as estimated activities were close to 0.

^f^ Allele frequency and allelic percentage activity estimate deviate from expectations and are further addressed in the discussion.

^g^ Allelic percentage activity of CNVs represents the relative increase of additional allele copies compared with the first copy.

**Table 3:** Final parameter estimates of the joint parent‐metabolite nonlinear mixed‐effects model of tamoxifen and endoxifen

	Parameter (unit)	Parameter description	Estimate (% CV)	RSE^j^, %
Fixed effects	F^a^, ^b^	Apparent bioavailability	1	Fixed
	Non‐adherence^c^	Fractional change in F due to non‐adherence	−0.726	1.57
	*k*_a_ (1/h)	Absorption rate constant	1.18	12.8
	*t*_lag_ (h)	Absorption lag time	0.401	15.3
	V_TAM,C_/F (L)	Tamoxifen volume of distribution	770	4.86
	CL_TAM_/F (L/h)^a^	Typical tamoxifen clearance	6.05	0.718
	Age^d^	Exponent for covariate effect of age on CL_TAM_/F	−0.269	11.5
	Body weight^d^	Exponent for covariate effect of body weight on CL_TAM_/F	0.170	21.2
	CYP3A4^e^	Exponential fractional change for covariate effect of CYP3A4 ≠ NM	0.163	21.2
	CL_CYP2D6_/F (L/h)^a^	Typical CYP2D6‐dependent Z‐endoxifen formation	0.478	1.79
	*CYP2D6* alleles^c^	Fractional change in CL_CYP2D6_/F due to *CYP2D6* genotype	See **Table** **2**	
	CYP2D6 inhibitor (weak/moderate)^c^	Fractional change in CL_CYP2D6_/F due to weak/moderate or strong CYP2D6 inhibitor	0.682	6.76
	CYP2D6 inhibitor (strong)^c^		0	Fixed
	CL_other_/F (L/h)^a^	Typical CYP2D6‐independent Z‐endoxifen formation	0.160	2.31
	CYP2C9^e^	Exponential fractional change for covariate effect of CYP2C9 ≠ NM	0.378	14.6
	CL_Z4′MET_/F (L/h)	Typical Z4′‐endoxifen formation from tamoxifen	0.315	1.22
	V_Z‐ENDX,C_/F (L)^f^	Z‐endoxifen volume of distribution	400	Fixed
	CL_Z‐ENDX_/F (L/h)^a^, ^f^	Typical Z‐endoxifen clearance	5.10	Fixed
	Body weight^d^	Exponent for covariate effect of body weight on CL_Z‐ENDX_/F	0.406	8.35
	V_Z4′‐ENDX,C_/F (L)^f^	Z4′‐endoxifen volume of distribution	400	Fixed
	CL_Z4′‐ENDX_/F (L/h)^a^, ^f^	Typical Z4′‐endoxifen clearance	5.10	Fixed
	Body weight^d^	Exponent for covariate effect of body weight on CL_Z4′‐ENDX_/F	0.234	26.4
Random effects	IIV CL_TAM_/F^g^	Interindividual variability in tamoxifen clearance	0.244 (52.6)	1.11
	IIV CL_CYP2D6_/F+ CL_other_/F^g^, ^h^	Interindividual variability in joint Z‐endoxifen formation	0.0755 (28.0)	3.84
	RUV TAM monosample^i^	Residual unexplained variability in observed tamoxifen concentrations for studies with one or multiple PK samples per patient	0.0852 (29.8)	2.44
	RUV TAM multisample^i^		0.0292 (17.2)	1.55
	RUV Z‐ENDX monosample^i^	Residual unexplained variability in observed Z‐endoxifen concentrations for studies with one or multiple PK samples per patient	0.175 (43.7)	2.18
	RUV Z‐ENDX multisample^i^		0.027 (16.5)	1.58
	RUV Z4′‐ENDX^i^	Residual unexplained variability in observed Z4′‐endoxifen concentrations	0.171 (43.2)	2.31
	RUV total ENDX^i^	Residual unexplained variability in observed total endoxifen concentrations	0.283 (57.2)	2.73

The reference patient had a body weight of 70 kg, age of 50 years, a CYP2D6*1/*1 genotype, CYP3A4 and CYP2C9 NM phenotypes, was considered adherent and received no CYP2D6 inhibitor co‐medication. Parameter estimates for *CYP2D6* alleles are summarized in **Table** **2**

CV, coefficient of variation; ENDX, endoxifen; IIV, interindividual variability; PK, pharmacokinetics; RUV, residual unexplained variability; TAM, tamoxifen.

^a^ Population estimates represent values obtained for the reference patient.

^b^ F was fixed to a hypothetical value of 1 as there were no data from intravenous administration available to support estimation.

^c^ Implemented as fractional change.

^d^ Implemented as power covariate model.

^e^ Implemented as exponential fractional change with ordinal scale (0: PM, 1: IM, 2: NM, 3: UM).

^f^ Parameters were fixed to values from the previously published pharmacokinetic model assuming similar PK of Z‐ENDX and Z4′‐ENDX as no data from metabolite administration were available to support estimation.

^g^ Implemented as exponential relation, representing a lognormal parameter distribution. Estimates are reported as variance (%CV).

^h^ IIV was added on joint Z‐ENDX formation (CL_CYP2D6_/F+ CL_other_/F).

^i^ Implemented as additive relation on the log‐scale, that is, exponential relation on the normal scale. Estimates are reported as variance (%CV).

^j^ RSEs were obtained from Sampling Importance Resampling.

#### Extension of PK NLME model

Z‐ENDX formation was split into a CYP2D6‐dependent and CYP2D6‐independent pathway, representing 74.9% and 25.1% of typical Z‐ENDX formation. An additional compartment for Z4′‐ENDX allowed the implementation of total ENDX (n = 961 samples) as sum of Z‐ENDX and Z4′‐ENDX. Including separate RUV models led to a 2.92‐fold and 6.48‐fold increase in RUV variance for patients with one vs. multiple sampling timepoints for TAM and Z‐ENDX, respectively, and was retained to avoid inflated IIV. Further changes to the structural, covariate, and RUV model are outlined in **Supplementary** **Text S2**.

#### Modeling *CYP2D6* ‐APA

*CYP2D6*‐APAs were estimated on a scale from 0% to 100%. *CYP2D6*‐APAs of nonfunctional CYP2D6*3, *4 and *6 alleles were fixed to 0% as estimates approached 0% with ≥50% RSE. In addition, very rare no function alleles (frequency <0.1%: CYP2D6*11, *12, *13, *15, *19, *21, *31, *36 (singleton), *40, *68 (singleton)) were fixed to 0% and very rare decreased (CYP2D6*14) and normal function (CYP2D6*34) alleles were fixed to their categorical AVs as the data did not support estimation. Initial *CYP2D6*‐APAs, not considering limited *CYP2D6* genotyping, are summarized in **Table** **2**. For CNVs, each additional allele copy was estimated to exhibit an activity of 68.4% compared with the first copy. This increase was smaller than postulated by categorical AVs, which multiply the AV of an allele by its copy number (e.g., ×2, ×3).^11^ Testing interaction between alleles, CYP2D6*1 CYP2D6‐APA was estimated to be 144% (14.4% RSE, 95% CI: 105%–187%) when in combination with CYP2D6*10 (n = 45 occurrences with Roche AmpliChip). Although exploratory, this was considerably higher than 100% *CYP2D6*‐APA (associated with additive interaction).

#### Modeling CYP2D6 co‐medication

Residual CYP2D6 activity under co‐medication with strong CYP2D6 inhibitors was fixed to 0% as the estimate approached 0% with ≥50% RSE, corresponding to complete CYP2D6 inhibition. Weak/moderate CYP2D6 inhibitors reduced CYP2D6 allele activity to 68.2% of their respective *CYP2D6*‐APA. Weak and moderate inhibitors were combined into one category due to the low number of patients taking moderate inhibitors (*n* = 7, **Table** **1**).

### Analysis of CYP2D6‐independent influential factors

The following CYP2D6‐independent covariates were added or retained in the final model: non‐adherence on TAM bioavailability, *CYP3A4* genotype‐predicted‐phenotype, age and weight on TAM clearance, *CYP2C9* genotype‐predicted‐phenotype on CYP2D6‐independent Z‐ENDX formation and weight on Z‐ENDX and Z4′‐ENDX clearance. All covariates were already included in the previously published model and a sensitivity analysis testing the impact of missing covariate information yielded comparable parameter estimates, confirming the robustness of the analysis (**Supplementary** **Text S2**). All tested candidate covariates and intermediate steps of covariate inclusion are detailed in **Supplementary** **Text S2**.

### Assessing bias in *CYP2D6* ‐APA through limited genotyping

*CYP2D6* allele frequencies from genotyping with Roche AmpliChip were stratified by White, Asian, and Black/African American patients (**Table** **S3**). When considering limited genotyping, some apparent CYP2D6*1 reference activities across genotyping assays deviated substantially from 100%, correlating with the percentage of alleles misclassified as CYP2D6*1 (**Figure** **S3**): With approximately 10% of alleles defaulting to CYP2D6*1, apparent CYP2D6*1 reference activity dropped to 90% or lower. Accordingly, *CYP2D6*‐APA was overestimated by 1.08‐fold (median, range: 1.05–1.14) if limited genotyping was neglected, corresponding to a more pronounced absolute change for alleles with higher activity (**Table** **2**).

Estimated TAM‐derived *CYP2D6*‐APAs were spread throughout the entire range from 0% to 100%. While some alleles aligned well with their categorical AV, others deviated considerably (**Figure** **3**, **Table** **2**). Several normal function alleles (CYP2D6*2, *35, *39) had lower estimated *CYP2D6*‐APAs of 64%, 68%, and 70% than their assigned categorical AV of 1. Decreased function alleles showed a wide range of estimated *CYP2D6*‐APAs (16%–52%): among alleles with a categorical AV of 0.25, CYP2D6*10 and *41 (23% and 16%) were similar to their assigned categorical AV, whereas CYP2D6*9 activity (52%) was closer to its original AV of 0.5 before being reclassified by CPIC. In contrast, CYP2D6*17 and *29 (categorical AV of 0.5) showed lower *CYP2D6*‐APAs of 29% each. CYP2D6*7 was the only no function allele yielding an appreciable yet exploratory *CYP2D6*‐APA of 16% as described in the discussion.

**Figure 3:**
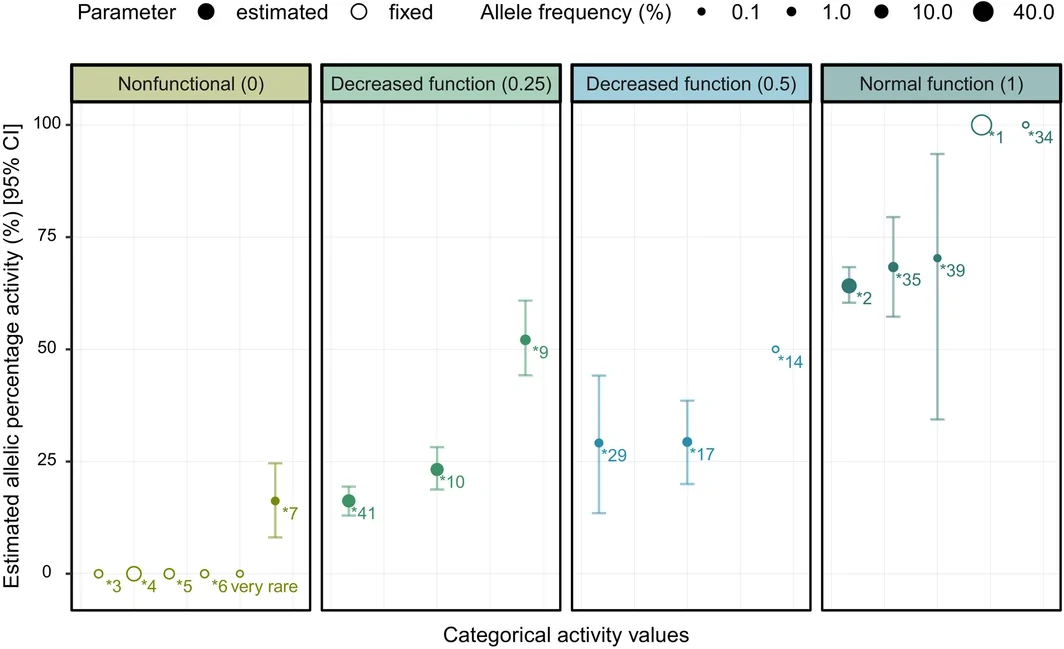
Final *CYP2D6* allelic percentage activities stratified by their respective categorical Clinical Pharmacogenetics Implementation Consortium (CPIC)‐assigned activity values. Solid circles and error bars represent estimated median (95% CI) final *CYP2D6* allelic percentage activities, stratified and colored by categorical allele activity value. Open circles represent fixed allelic percentage activities due to low allele frequency or estimates close to 0. Circle size represents allele frequency.

Accordingly, individual *CYP2D6*‐PAs of CEPAM patients differed substantially from their assigned CYP2D6 AS: *CYP2D6*‐PAs predicted overall lower CYP2D6 activity and showed considerable variability within AS categories (**Figure** **4** **a**).

**Figure 4:**
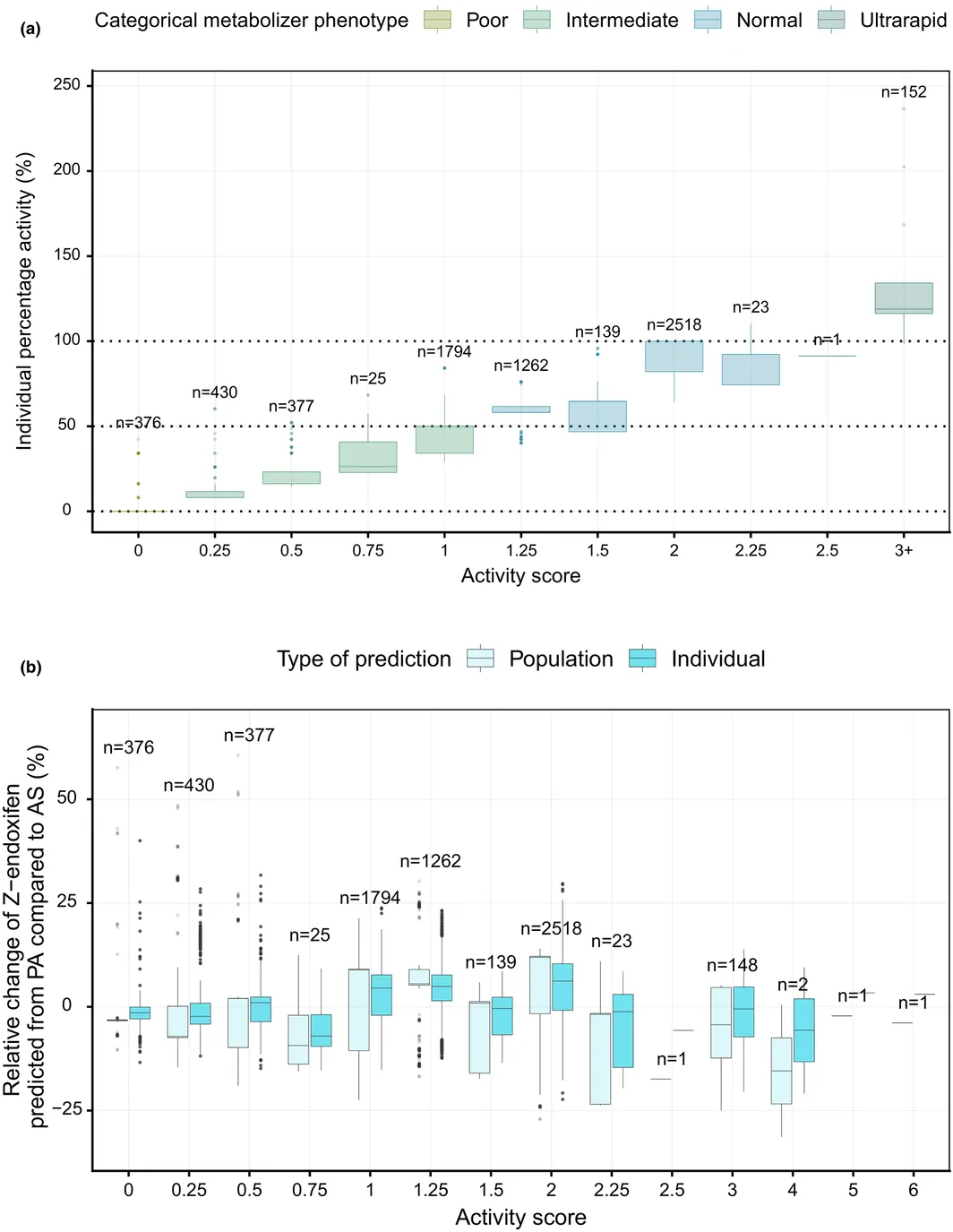
Final individual *CYP2D6* percentage activities and their impact on Z‐endoxifen concentrations compared with CYP2D6 activity score. (**a**) Final individual *CYP2D6* percentage activities stratified by CYP2D6 activity score obtained from Clinical Pharmacogenetics Implementation Consortium (CPIC)‐assigned activity values. Boxes represent the interquartile range (IQR) and median of individual *CYP2D6* percentage activities, whiskers are the range from first/third quartile to lowest/highest value within 1.5 IQR, and dots display values outside this range. The color represents the metabolizer phenotype (poor, intermediate, normal, and ultrarapid) assigned based on the activity score. Horizontal lines at percentage activities 0%, 50%, and 100% indicate the equivalent to categorical activity scores of 0, 1, and 2, respectively. The number of patients in each activity score group is shown. (**b**) Relative change in minimal Z‐endoxifen concentration predicted using CYP2D6 activity score and *CYP2D6* percentage activity. Relative change in population and individual predictions of minimal steady‐state Z‐endoxifen concentration at 24 hours after last tamoxifen administration obtained for CYP2D6 Endoxifen Percentage Activity Model (CEPAM) patients using *CYP2D6* percentage activity (*CYP2D6*‐PA) compared with CYP2D6 activity score (AS). Population predictions were based on population model parameters and baseline patient covariates, while individual predictions were based on their individual model parameters and baseline patient covariates. Boxes represent the interquartile range (IQR) and median of individual changes, whiskers are the range from hinge to lowest/highest value within 1.5 IQR, and dots display values outside this range. The number of patients in each CYP2D6 AS group is shown.

### Assessment of impact of clinical covariates on Z‐ENDX

Included covariates reduced total IIV on Z‐ENDX formation in the final model from 57.7% CV to 28.0% CV (2.06‐fold). *CYP2D6*‐PA alone reduced IIV on Z‐ENDX formation by 27.3% CV. Directly compared with the currently established categorical AV, *CYP2D6‐*PA explained slightly more IIV in Z‐ENDX formation (1.12‐fold reduction). The impact of CYP2D6‐independent covariates and covariate combinations additionally supported the importance of CYP2D6 activity for the case example of TAM and Z‐ENDX (**Figure** **S4**, **Supplementary** **Text S2**). Importantly, differences observed between individual *CYP2D6*‐PAs and assigned CYP2D6 AS (**Figure** **4** **a**) translated to changes in Z‐ENDX concentrations (**Figure** **4** **b**): Predicted population and individual minimal steady‐state Z‐ENDX concentrations 24 hours after TAM administration using either *CYP2D6*‐PAs or assigned CYP2D6 AS showed relative changes ranging from −31.4 to 60.6% and −22.3% to 40.0%, respectively.

## DISCUSSION

CYP2D6 plays a major role in the metabolism of many frequently prescribed drugs. However, the current translation from *CYP2D6* genotype to predicted CYP2D6 phenotype relies on an imprecise system of categorical AVs, which may lead to phenotype misclassification and potential under‐/overdosing. A more precise *CYP2D6*‐PA system could improve individualized dosing and treatment outcomes for CYP2D6 substrates. We therefore developed an NLME framework assessing *CYP2D6*‐APA for TAM as model substrate on an intuitive 0% to 100% scale based on data from >7,000 patients from a global multi‐study database. Several estimated TAM‐derived *CYP2D6*‐APAs deviated substantially from their assigned categorical AVs, highlighting the relevance of refining the *CYP2D6* genotype‐to‐phenotype translation system to optimize treatment recommendations and therapeutic outcomes.

To our knowledge, this analysis represents the most comprehensive assessment of clinical *CYP2D6*‐APA to date. It included the most frequent alleles recommended for clinical testing by the Association for Molecular Pathology (AMP) and other leading organizations,^16^ thus expanding previous analyses of *CYP2D6*‐APA.^13^, ^14^, ^15^ All 10 *CYP2D6* star alleles categorized as AMP Tier 1 (must for clinical genetic testing, **Table** **2**) were included in the present analysis, as well as seven out of the 12 Tier 2 alleles. Moreover, the global nature of the CEPAM database ensured representation of alleles prevalent only among some biogeographical groups,^17^ such as CYP2D6*17 or *29 in patients of African ancestry.^29^

In our analysis, several TAM‐derived *CYP2D6*‐APAs deviated substantially from their CPIC‐assigned categorical AVs, highlighting the potential benefit of more precise genotype‐to‐phenotype translation. Similar results were reported in single‐study analyses of TAM and Z‐ENDX.^14^, ^15^, ^30^ Although all cohorts were included in the CEPAM database, a comparison is of interest due to the variety of applied methodologies: linear regression,^30^ pharmacometric modelling,^15^ and a neural network of full *CYP2D6* gene sequencing.^14^ Of note, the methodology applied in this work leveraging NLME modeling is similar to a previously proposed approach by Agema et al.^15^ In addition to Agema et al., the present analysis accounted for limited genotyping and expanded its scope with broad allele coverage from diverse populations. Moreover, multiple studies evaluated *CYP2D6* allele activity for other substrates,^13^, ^31^, ^32^, ^33^, ^34^, ^35^, ^36^, ^37^ such as venlafaxine, risperidone, aripiprazole, or dextromethorphan. **Table** **S4** provides a comparison between *CYP2D6*‐APA estimates reported across different substrates. Future work should rigorously compare *CYP2D6*‐APA estimates derived from various substrates to determine whether, and for which alleles, substrate‐specific *CYP2D6*‐APA systems are needed to further advance precision dosing.^38^

Several alleles categorized as normal function (AV = 1) demonstrated decreased CYP2D6 activity relative to the CYP2D6*1 reference allele in this analysis, such as CYP2D6*2 (64%) and *35 (68%). Decreased CYP2D6*2 activity has been reported across multiple studies with various substrates.^14^, ^15^, ^30^, ^31^, ^35^, ^36^, ^37^, ^39^ Differences between activity estimates (10%–76%, **Table** **S4**) may be explained, at least partially, by substrate specificity, varying methodologies, limited genotyping, or star allele subvariants with different activity.^36^, ^40^ Considering all available data, results indicate that CYP2D6*2 has less activity than *1 and that the degree of reduction may be substrate‐dependent. Similar point estimates were obtained for CYP2D6*35 activity,^14^, ^36^ although with larger CIs due to lower sample sizes.

*CYP2D6*‐APAs of decreased function alleles were spread across a wide range of 16% to 52% in this analysis. For CYP2D6*10 and *41, estimated *CYP2D6*‐APAs of 23% and 16% aligned well with their assigned AV of 0.25, as well as with estimates across all evaluations with slight differences between substrates.^13^, ^14^, ^15^, ^31^, ^32^, ^33^, ^34^, ^36^ In contrast, while CYP2D6*17 and *29 APA estimates (each 29%) deviated moderately from their AV of 0.5, multiple evaluations showed differences between substrates and wide CIs.^15^, ^30^, ^31^, ^32^, ^37^, ^39^ Plausible explanations include low sample sizes and substrate specificity, as mentioned for CYP2D6*17 based on *in vitro* experiments and *in vivo* data.^37^, ^39^, ^41^ Notably, Kehinde et al. and Bennett et al.^37^, ^39^ found increased activity of CYP2D6*17 toward risperidone, suggesting profound substrate‐specific activity. Lastly, CYP2D6*9 showed considerable differences between the *CYP2D6*‐APA estimate of 52% and the current AV of 0.25. Of note, CYP2D6*9 was reclassified from a categorical AV of 0.5 to 0.25 based on published literature available at the time. Our analysis, based on 313 occurrences, suggests that the previous classification of 0.5 may be more appropriate for TAM, and perhaps other substrates. Previous reports demonstrated higher activity for CYP2D6*9 compared with *10 for TAM, risperidone, and tedatioxetine,^14^, ^32^, ^36^ while others observed no difference between these alleles for venlafaxine, risperidone or aripiprazole.^13^, ^34^ Comprehensive *in vivo* and *in vitro* evaluations are required to confirm substrate‐specific *CYP2D6*‐APAs for CYP2D6*9 and clinical implications on precision dosing.

Among the no function alleles (AV = 0), the rather rare CYP2D6*7 allele had an estimated *CYP2D6*‐APA of 16.2% (95% CI: 8.10%–24.6%). CYP2D6*7 has an amino acid substitution near the active site disrupting protein folding and believed to result in a nonfunctional enzyme.^42^ Of note, allele frequency (n = 48, 0.338%) was higher than expected (Total: 0.0782%, Black/African American 0.00538%^43^), especially for Black/African American patients (*n* = 34, 3.77%), originating from two out of 36 studies, potentially raising concerns regarding genotype test accuracy in these cases. While a sensitivity analysis excluding CYP2D6*7 confirmed no impact on other estimated *CYP2D6*‐APAs, the observed cyp2d6*7 apa should be interpreted with caution.

The effect of additional allele copies was estimated to be 68.4% of the first copy, which, if confirmed across substrates, is significantly lower than the doubling of activity assumed by the AS system. This estimate relied on the assumption that all copies were the same allele and that additional copies showed additive allele activity, which may not reflect genetic complexity; the limited frequency of CNV in our analysis dataset (2.05%) did not allow for more detailed analysis, requiring a dedicated study for further assessment. In addition, potential interactions between *CYP2D6* alleles were evaluated: The *CYP2D6*‐APA of CYP2D6*1 was estimated to be 144% (95% CI: 105%–187%) when in combination with CYP2D6*10. Although based on only 45 diplotype occurrences, the estimate and 95% CI were considerably higher than 100%, suggesting non‐additive allele interactions, for example, due to protein–protein interactions between both isoforms.^44^ While these findings could impact the established assumption of additive allele activity in CYP2D6 AS and PA, dedicated studies across alleles and substrates are needed.

To inform unbiased estimates of *CYP2D6*‐APA, limited genotyping was considered. Various authors postulated that limited allele coverage in genotyping assays may be problematic due to misclassified alleles defaulting to CYP2D6*1.^16^, ^22^, ^23^ Our analysis revealed that alleles misclassified as CYP2D6*1 can cause considerably lower apparent CYP2D6*1 activity, resulting in biased *CYP2D6*‐APAs, as these are usually normalized to CYP2D6*1 activity. Nonetheless, no previous analysis of *CYP2D6*‐APA considered this effect, despite misclassification risk from limited genotyping in some analyses (e.g., CYP2D6*2 not tested).^13^, ^34^ While our analysis provided a suitable framework to consider limited genotyping, it assumed that all misclassified alleles defaulted to CYP2D6*1. However, some genotyping assays only test for a limited set of variants, and alleles may be defaulted to other star alleles as well. For instance, CYP2D6*10 shares a single nucleotide variation (100C>T) with ≥15 less common alleles, which may be wrongly called as CYP2D6*10 if their unique variants are not tested.^16^ However, in contrast to alleles defaulted to CYP2D6*1, default assignments to other alleles only affect the *CYP2D6*‐APA estimate for the respective default allele and not the whole PA scale. In addition, in the case of CYP2D6*10, all potentially misclassified alleles are very rare, hence minimal changes would be expected.

This analysis also considered CYP2D6‐independent covariates to estimate *CYP2D6*‐APA. CYP2D6 phenotype is not solely dependent on *cyp2d6* genotype: Patient‐ and disease‐related factors, such as co‐medication(s), age, pregnancy, or inflammation can cause phenoconversion.^45^ In fact, we showed that weak/moderate CYP2D6 inhibitors reduce enzymatic activity by 32%, while strong inhibitors caused complete abolishment. Moreover, various factors have been described to contribute to variability in transcriptional *CYP2D6* regulation,^46^, ^47^ but none has emerged as a biomarker for phenotype prediction and they are typically not used in clinical practice. Our analysis did account for many genetic and clinical factors previously reported to affect metabolic activation of TAM to Z‐ENDX, including age, body weight, *CYP2C9* and *CYP3A4* genotype.^15^, ^21^, ^48^ Of note, large amounts of missing data were reported for some covariates, for example, genotype‐predicted‐phenotypes for CYP2C9 and CYP3A4, calling for cautious interpretation of these findings. Nonetheless, a sensitivity analysis supported the robustness of our analysis (**Supplementary** **Text S2**). Other reported factors, including CYP2C19,^21^ CYP2B6,^21^ or CYP3A4 inducers,^20^ were not confirmed in this comprehensive analysis, potentially due to small effect size or insufficient data. While the model generally predicted the observed concentrations well, there was a small underprediction observed for high TAM and ENDX concentrations. Due to the consistent pattern between both analytes, this trend was expected to result from a minor misspecification of the TAM model. Given the large size of the dataset, the parsimonious model structure (one compartment per analyte), and the other goodness‐of‐fit plots indicating adequate model performance (**Figure** **2** **c**), this trend was deemed acceptable. Since these underpredictions were isolated to high concentrations, no impact on the conversion of TAM into Z‐ENDX, and consequently on *CYP2D6*‐APA estimates, was expected.

This *CYP2D6*‐APA model was generated for the case example of TAM, using the large international CEPAM cohort. Current CPIC guidelines recommend doubling the TAM dose, or switching to aromatase inhibitors for CYP2D6 IM/PM patients, based on the principle of exposure‐matching following strong evidence indicating lower individual systemic Z‐ENDX concentrations at the standard dosing of 20 mg/day.^6^ However, while several Z‐ENDX minimal plasma concentrations were proposed as efficacy thresholds, none have been validated in prospective clinical trials.^49^ The conduct of new prospective trials with sufficient sample size and follow‐up time to assess an exposure‐response relationship for Z‐ENDX is unlikely for an old drug like TAM. Therefore, planned future work will apply this *CYP2D6*‐PA model to re‐analyze survival outcomes in large prospective studies without measured PK data, including the International Tamoxifen Pharmacogenetics Consortium (ITPC) dataset.^49^ Should such future work confirm an exposure‐response relationship for Z‐ENDX, simulations based on *CYP2D6*‐PA could put such thresholds into context and support refined CPIC recommendations. Of note, predicted Z‐ENDX concentrations based on the AS vs. *CYP2D6*‐PA systems showed individual differences ranging between −22.3% and 40.0%. Although indicating the potential for precision dosing, *CYP2D6*‐PA may not immediately translate to improved dosing recommendations for TAM due to considerable remaining IIV, in alignment with the only modest increase of explained IIV (1.12‐fold) of *CYP2D6*‐PA compared with the categorical AS. Indeed, a simulation study by Centanni et al. showed that for TAM, Therapeutic Drug Monitoring, that is, dose adaptation based on measured drug concentrations, might outperform pharmacogenomic‐based dosing, while pharmacogenomic‐based dosing was superior for drugs with small remaining IIV.^50^

Beyond TAM, the developed *CYP2D6*‐PA system may have broad benefit across CYP2D6 substrates, especially medications for which CPIC guidelines already exist.^2^, ^3^, ^4^, ^5^, ^6^, ^8^, ^9^ Precise *CYP2D6*‐APA estimates obtained for the model substrate TAM in this work provide a robust foundation for evaluating the *CYP2D6*‐PA system across substrates. Replacing naïve categorical AVs with empirically estimated *CYP2D6*‐APAs may be more intuitive to clinicians and could be directly translated into precise dosing recommendations (e.g., a patient with 60% *CYP2D6*‐PA should receive 60% of the standard dose of a CYP2D6‐eliminated drug). Medications with narrow therapeutic windows and strong exposure‐outcome relationships will benefit most from precise metabolic activity estimation and dosing recommendations, especially when fine‐grid dosing options (e.g., infusions and oral medications with small dose strength steps/flexible dosing intervals) are available. Moreover, understanding the *CYP2D6*‐APA of *CYP2D6* alleles with particularly variable activity across substrates may become an important objective in clinical development of new drugs metabolized by CYP2D6, and allow the identification of patient subpopulations at higher risk of under‐ or overdosing early‐on. Of note, updating *CYP2D6* genotype‐to‐phenotype translation systems will introduce logistical challenges to laboratories conducting testing and sites implementing pharmacogenetics in clinical practice.

To successfully evolve from AS to PA systems beyond TAM, substrate specificity of allelic metabolic activity must be considered, as precise *CYP2D6*‐APA estimates for one substrate may not apply to others (**Table** **S4**). Importantly, the TAM‐derived *CYP2D6‐*APA estimates should not be applied to other substrates unless comprehensively validated. Joint efforts similar to the CEPAM consortium, enabling pooled multi‐study evaluations, are needed to rigorously assess *CYP2D6*‐APA across the plethora of alleles and substrates to determine whether substrate‐specific *CYP2D6*‐PAs are needed. If substrate‐specific *CYP2D6‐*APAs are required, they could be obtained following the framework proposed in this work. Similarly, although the *CYP2D6*‐PA system was developed for CYP2D6, the framework can be applied to any other enzyme, and potentially other pharmacogenomics associations, to improve precision and intuitiveness of pharmacogenomics‐guided treatment. To robustly estimate APAs for common variants of other pharmacogenes, additional research using large clinical cohorts with PK data, ideally supplemented with *in vitro* or other *in vivo* data, is needed.

In conclusion, this analysis utilized NLME modeling of the large, comprehensive CEPAM database to develop a robust *CYP2D6*‐PA system. These newly derived *CYP2D6*‐APA estimates will contribute to a more precise understanding of the quantitative impact of *CYP2D6* genetics in drug metabolism. These results lay the foundation for updating the *CYP2D6* genotype‐to‐phenotype translation system and, upon prospective validation of its effect on drug exposure and clinical outcomes, may inform more precise dosing and optimize therapeutic outcomes across CYP2D6 substrates.

## FUNDING

This work was in part funded by the Western Norwegian Health Authority Grant number: F‐10450 and the US–Norway Fulbright Foundation. W.S. and M.S. are in parts supported by the Robert Bosch Stiftung Stuttgart, Germany. M.S. is funded by the Deutsche Forschungsgemeinschaft (DFG, German Research Foundation) under Germany's Excellence Strategy—EXC 2180‐390900677.

## CONFLICT OF INTEREST

C.K. reports research grants from an industry consortium (AbbVie, AstraZeneca, Boehringer Ingelheim, F. Hoffmann‐La Roche, Merck KGaA, Novo Nordisk, Sanofi) for the graduate research training program PharMetrX and from the H2020‐EU.3.1.3 (“FAIR”), all outside the submitted work. D.L.H. reports support unrelated to this study but provided to his institution in the last 24 months during conduct and analysis of this study from LaCar MDX Technologies. A.M.M. is an employee of Pharmetheus AB and a paid consultant to multiple pharmaceutical companies. All other authors declared no competing interests for this work. As an Editor‐in‐Training for *Clinical Pharmacology & Therapeutics*, Anna Mc Laughlin was not involved in the review or decision process for this paper.

## AUTHOR CONTRIBUTIONS

C.K., D.L.H., and F.K. designed the research. F.K., T.H., and R.M. performed the research. F.K. analyzed the data. All authors wrote the manuscript.

## Supporting information


Supplementary Text S1.

Supplementary Text S2.

Figure S1.

Table S1.
