# Supplementary material for: A Precise Activity System for Pharmacogenetics Based on Estimation of cyp2d6 allelic Percentage Activity from the International CEPAM Database
Source: Clin Pharmacol Ther. 2026 Nov 11:10.1002/cpt.70560. Online ahead of print. doi: 10.1002/cpt.70560 (PMC13755603; doi:10.1002/cpt.70560)
Supplement: Supplementary file 1 [file CPT-9999-0-s001.docx]

**Supplementary figures**

**Figure S1** Proportion of missing data for patients/samples from studies with one vs. multiple sampling times per patient. For time after dose and adherence, missing data was assessed on the sample level. For all other covariates, missing data was assessed on the patient level at baseline.

**Figure S2** Additional goodness-of-fit (GOF) plots and visual predictive checks (VPC) for the final extended model. (**a**) GOF plots for the final extended model comparing typical (left) and individual (right) tamoxifen (TAM) predictions with the measured concentrations. Predictions and concentrations are dose-normalized to 20 mg TAM dosing. The diagonal black line marks the line of identity. (**b**) Diagnostic plots of conditional weighted residuals (CWRES) for TAM predictions vs. typical TAM predictions (left) and time after last dose (right) using the final extended model. The black horizontal lines mark the zeroline and CWRES of +5 and -5. (**c**) Prediction-corrected VPC for TAM (top) and Z-ENDX (bottom) based on 200 simulations including interindividual variability as well as residual unexplained variability. Black solid lines: observed medians. Blacked dashed lines: 5th and 95th percentile of the observed values. Red or blue solid lines: simulated medians. Red or blue dashed lines: simulated 5th and 95th percentiles of the simulated values. Red or blue shaded areas: 95th confidence intervals (CI) around the simulated medians, 5th and 95th percentiles.

**Figure S3** Apparent *CYP2D6*1* reference activity vs. proportion of alleles misclassified as *CYP2D6*1* per genotyping assay used in the CEPAM analysis dataset.

**Figure S4** Forest plot illustrating the effect of influential factors on minimal Z-endoxifen (Z-ENDX) concentrations in steady-state. Values were normalized to predictions of a reference patient, illustrated by the black dotted vertical line. The gray area represents the exposure range for bioequivalence (0.8-1.25-fold of reference). Blue points represent the median predicted relative change in Z-ENDX concentration compared to the reference patient and blue lines are 90% confidence intervals (CI). Specific values of median and 90% CI are summarized in the box on the right. The reference patient was female with a *CYP2D6*1/*1* genotype, no CNV, no CYP2D6 inhibitor co-medication, adherent to treatment, normal metabolizer (NM) for CYP3A4 and CYP2C9, 50 years of age and a body weight of 70 kg. All illustrated cases apart from the worst/best-case combinations represent univariate changes of one covariate at a time. The following combinations of covariates were selected for worst-case combinations with *weak*, *moderate*, and *strong impact*: Compared to the reference patient, the *weak impact* combination represented a patient with normal CYP2D6 activity and covariates with moderate covariate effect, i.e., intermediate metabolizer (IM) for CYP3A4, a poor metabolizer (PM) for CYP2C9, aged 36 years, and weighing 97 kg. Compared to the *weak impact* combination, the *moderate impact* combination represented a patient with additionally decreased CYP2D6 activity, i.e., a *CYP2D6*41/*41* genotype and weak/moderate CYP2D6 inhibitor co-medication. Compared to the *weak impact* combination, the *strong impact* combination represented a patient who additionally was non-adherent and had no CYP2D6 activity, i.e., a *CYP2D6*5/*5* genotype, strong CYP2D6 inhibitor co-medication. The following combinations of covariates were selected for the best-case combination: Compared to the reference patient, the *strong impact* combination had a genotype *CYP2D6*1x2/*1x2* genotype, aged 71 years and weighing 52 kg.
