# Supplementary material for: A Precise Activity System for Pharmacogenetics Based on Estimation of cyp2d6 allelic Percentage Activity from the International CEPAM Database
Source: Clin Pharmacol Ther. 2026 Nov 11:10.1002/cpt.70560. Online ahead of print. doi: 10.1002/cpt.70560 (PMC13755603; doi:10.1002/cpt.70560)
Supplement: Supplementary file 2 [file CPT-9999-0-s002.docx]

**Supplementary Text S1: Supplementary Methods**

**Clinical study database**

During data cleaning, patients or studies were assessed for (1) eligibility for analysis, (2) distribution of TAM and ENDX measurements and (3) plausibility of reported dosing or pharmacokinetic data as previously described^1^:

(1) Patients were not eligible for analysis and were excluded if they were male, had not reached TAM steady state (treatment duration of <1 month), had missing/unclear *CYP2D6* genotype or missing TAM or ENDX concentrations.
(2) Studies were excluded from the analysis if they had considerably higher or lower TAM or ENDX concentrations compared to the rest of the studies upon graphical and numerical inspection and no explanatory factors as described previously^1^.
(3) Patients were excluded if they had sampling times after last dose of zero or >48h, a reported TAM dose of 1 mg or implausible reported TAM or ENDX concentrations.

Studies with considerably higher Z-ENDX or lower total ENDX concentrations compared to the other studies with the respective ENDX analyte were reassigned to total ENDX or Z-ENDX, respectively, if the review of the bioanalytical method supported this, as previously described in detail^1^.

Missing data was imputed in the raw database after exclusion of male patients to avoid bias. Missing covariates were imputed as median of a reference group for continuous covariates and as most frequent category of a reference group for categorical covariates. If no correlation with other covariates was expected, the reference group was either the respective study for single missing values or the analysis dataset for missing data of a whole study. If correlations with other covariates were expected (e.g., biogeographical group), the reference group was the respective stratification group.

Missing times after last dose were imputed as 23h if only one sample was taken in a dosing interval and the study protocol stated that trough samples were obtained.

**Development of *CYP2D6* allelic percentage activity (*CYP2D6*-APA)**

**Extension of PK NLME model.** During model extension, the structural model, the covariate model and the residual variability (RUV) model were refined:

The structural model was extended to allow for implementation of *CYP2D6* allelic percentage activity (*CYP2D6*-APA): First, the implementation of total ENDX was changed from a conversion factor between Z-ENDX and total ENDX to a semi-mechanistic approach by adding Z4’-ENDX to the model: Z4’-ENDX was formed from TAM in a first-order process (CL_Z4’MET_/F) and eliminated from the central compartment V_Z4’-ENDX,c_/F with first-order clearance CL_Z4’-ENDX_/F. Since no data or literature from Z4’-ENDX administration was available, Z4’-ENDX formation CL_Z4’MET_/F and volume of distribution V_Z4’-ENDX,c_/F were fixed to values for Z-ENDX from the previously published PK model^1^ assuming similar PK of Z-ENDX and Z4’-ENDX. Total ENDX was then modeled as the sum of Z-ENDX and Z4’-ENDX, thus allowing to independently assess covariates on Z-ENDX and Z4’-ENDX formation. Second, the estimation of previously unidentifiable structural parameters was investigated. Third, upon implementation of *CYP2D6-*PA, the formation of Z-ENDX from TAM was split into a CYP2D6-dependent and a CYP2D6-independent pathway. This aimed to reflect the two Z-ENDX formation pathways and allowed CYP2D6-dependent Z-ENDX formation to drop to zero in case of nonfunctional enzyme.

The covariate model was refined as follows: Covariates were removed and re-evaluated later-on if they were affected by changes in the structural model or if new data was available to update their implementation. This was especially relevant for covariates based on surrogate markers, i.e., drug concentration as a marker for non-adherence, if the expanded CEPAM analysis dataset contained new data allowing for a re-assessment during covariate analysis.

The RUV model was extended as follows: For each considered analyte, i.e., TAM and Z-ENDX, exponential RUV models implemented as additive on the log-scale were evaluated with separate parameters for studies with a single versus multiple sampling time points. This error model was motivated by the high proportion of missing sample collection times in studies with a single PK observation (Figure S1), expected to result in greater unexplained variability compared to studies with multiple sampling times and fewer missing sample collections times (Figure S1). Allowing higher RUV in monosample studies prevented inflation of the estimated interindividual variability (IIV), ensuring that IIV was primarily informed by individuals with multiple samples. By separating the RUV for single- from multi-sampling studies, the unexplained variability introduced by missing time after last dose data was appropriately attributed to the RUV of monosample studies rather than to population-level IIV.

**Modeling *CYP2D6*-APA.** During implementation of *CYP2D6*-APA, *CYP2D6*-APAs were averaged to obtain diplotype activity, i.e. individual percentage activity (*CYP2D6*-PA), assuming no interaction between alleles. To challenge this assumption, diplotypes genotyped with Roche AmpliChip having a *CYP2D6*1* allele in combination with a decreased function *CYP2D6*10* allele were selected. *CYP2D6*10* had sufficient frequency in the dataset and the variant produces a protein which might structurally interact, i.e., form dimers, with protein encoded by *CYP2D6*1*. Thus, for *CYP2D6*1/*10* diplotypes, the activity of *CYP2D6*1* was estimated separately using the final model (including bias through limited genotyping) and evaluated whether it differed from 100%. All other parameters were fixed.

**Analysis of CYP2D6-independent influential factors**

Continuous covariates were normalized to round values closest to the median value in the dataset, i.e., 50 years for age and 70 kg for bodyweight, and tested for inclusion using a power relation. As previously published^1^, *CYP3A4* and *CYP2C9* genotype data was translated to genotype-predicted phenotypes^2,3^. Following the principle of parsimony to reduce the number of parameters to estimate and as previously described^1^, these genotype-predicted-phenotypes were implemented using an exponential ordinal relation centered around the normal metabolizer. The same approach without centering was adopted for enzyme inhibitor or inducer co-medication if the strength of the inhibiting/inducing effect was reported. Non-adherence was implemented as fractional change on TAM bioavailability if patients were reported as non-adherent in the CEPAM database.

After stepwise covariate modelling, a sensitivity analysis was carried out to assess the impact of covariate imputation on the estimated covariate effect by removing patients with imputed covariate data and re-estimating model parameters.

**Assessing bias in *CYP2D6*-APA through limited genotyping**

In addition to missing genotyping of alleles, duplications or multiplications may also not have been tested. These include the *CYP2D6*1xN*, **2xN, *4xN* and **41xN* copy number variants^4^. Two cases were considered:

(1) Incomplete genotype data for both the allele of interest and gene multiplication, i.e., true allele and CNV unknown: e.g., genotyping test did not include *CYP2D6*2* or **2xN*, i.e., both were assumed to be defaulted to *CYP2D6*1*.
(2) Identification of the allele of interest but not of gene multiplication, i.e., only CNV unknown, leading to the defaulting of the undetected CNV to its respective allele: e.g., genotyping test included *CYP2D6*2*, but not **2xN*, i.e., *CYP2D6*2xN* were defaulted to *CYP2D6*2*.

The third possible scenario, i.e., CNV present but unknown which allele was duplicated (e.g., *CYP2D6*1x2/*2* vs. **1/*2x2*, or **2x2/*4* vs. **2/*4x2*), was not considered since these patients were excluded from the analysis due to missing/unclear *CYP2D6* genotyping data. For case (1), the same approach was applied as described in the main text and Equation 3. However, the definition of *CYP2D6*-APA (*APA*) and allele frequency *freq* varied slightly: *APA* represented the expected *CYP2D6*-APA of both the allele and CNV, as outlined in Equation 1, while *freq* was the total allele frequency (as the presence of CNV did not increase allele frequency). For case (2), the same approach was applied, however the default allele was the same allele as the duplication, so for the example of *CYP2D6*2*, Equation 3 was adapted as follows:

$$\begin{aligned} {APA}_{*2,apparent}=\frac{{APA}_{*2}*{freq}_{*2}+ {APA}_{*2}*{freq}_{*2}*{freq}_{CNV}*SF}{{freq}_{*2}}, \#\left( 1 \right) \end{aligned}$$

with *freq_*2_* as *CYP2D6*2* allele frequency, *freq_CNV_* as CNV frequency and scaling factor *SF* constrained between 0 and 1.

**Assessment of impact of clinical influential factors on Z-ENDX**

To assess clinical significance of a covariate effect, the magnitude of interindividual variability (IIV) explained by each covariate was identified: Covariates were univariately removed from the final model, the increase in IIV summed up across all covariates and the absolute and relative reduction in IIV by each covariate quantified. Moreover, the magnitude of IIV explained by CYP2D6-PA compared to the categorical activity score was assessed in the same way.

To assess the effect of each covariate and covariate combinations on minimal Z-ENDX concentrations compared to a reference scenario, scenarios with changing covariates were defined and stochastic simulations (n=1000) carried out taking into account parameter uncertainty, but not interindividual variability and residual variability. The reference patient was a female with a *CYP2D6*1/*1* genotype, no CNV, no CYP2D6 inhibitor co-medication, adherent to treatment, normal metabolizer (NM) for CYP3A4 and CYP2C9, 50 years of age and a body weight of 70 kg. Several scenarios with univariately changing covariates were defined as follows: Compared to the reference patient, continuous covariates were changed to the 5^th^ and 95^th^ percentile in the dataset, all categories of categorical covariates present in the dataset were tested, and all relevant *CYP2D6* alleles were tested in combination with *CYP2D6*1*. In addition, the following combinations of covariates were selected for worst-case combinations with *weak*, *moderate*, and *strong impact*: Compared to the reference patient, the *weak impact* combination represented a patient with normal CYP2D6 activity and covariates with moderate covariate effect, i.e., intermediate metabolizer (IM) for CYP3A4, a poor metabolizer (PM) for CYP2C9, aged 36 years, and weighing 97 kg. The *moderate impact* combination represented a patient with decreased CYP2D6 activity, i.e., a *CYP2D6*41/*41* genotype and weak/moderate CYP2D6 inhibitor co-medication, in addition to the *weak impact* combination. Finally, the *strong impact* combination represented a non-adherent patient with no CYP2D6 activity, i.e., a *CYP2D6*5/*5* genotype, strong CYP2D6 inhibitor co-medication, in addition to the *weak impact* combination. The following combinations of covariates were selected for the best-case combination: Compared to the reference patient, the *strong impact* combination had a genotype *CYP2D6*1x2/*1x2* genotype, aged 71 years and weighing 52 kg.

**Model performance and software**

Nested models were compared based on difference in objective function values (likelihood ratio test) while for non-nested models, difference in Akaike information criterion was used. Parameter precision was obtained from the NONMEM covariance step for intermediate models and using sampling importance resampling for the final model and all presented relative standard errors (unless specifically stated otherwise) and confidence intervals.

For modelling and simulation activities, NONMEM v7.5.1 was used via PsN (Perl Speaks NONMEM) v4.7.0. Data management, data visualization and postprocessing of modeling and simulation results were performed using R v4.4.0 with RStudio v2024.04.0.

**References**

1.	Mc Laughlin, A. M. *et al.* Nonlinear Mixed-Effects Model of Z-Endoxifen Concentrations in Tamoxifen-Treated Patients from the CEPAM Cohort. *Clin. Pharmacol. Ther.* **116**, 690–702 (2024).

2.	Pratt, V. M. *et al.* CYP3A4 and CYP3A5 Genotyping Recommendations: A Joint Consensus Recommendation of the Association for Molecular Pathology, Clinical Pharmacogenetics Implementation Consortium, College of American Pathologists, Dutch Pharmacogenetics Working Group of the Royal Dutch Pharmacists Association, European Society for Pharmacogenomics and Personalized Therapy, and Pharmacogenomics Knowledgebase. *J. Mol. Diagn.* **25**, 619–629 (2023).

3.	Whirl-Carrillo, M. *et al.* An Evidence-Based Framework for Evaluating Pharmacogenomics Knowledge for Personalized Medicine. *Clin. Pharmacol. Ther.* **110**, 563–572 (2021).

4.	Pratt, V. M. *et al.* Recommendations for Clinical *CYP2D6* Genotyping Allele Selection: A Joint Consensus Recommendation of the Association for Molecular Pathology, College of American Pathologists, Dutch Pharmacogenetics Working Group of the Royal Dutch Pharmacists Association, and the European Society for Pharmacogenomics and Personalized Therapy. *J. Mol. Diagn.* **23**, 1047–1064 (2021).
