# Supplementary material for: A Precise Activity System for Pharmacogenetics Based on Estimation of cyp2d6 allelic Percentage Activity from the International CEPAM Database
Source: Clin Pharmacol Ther. 2026 Nov 11:10.1002/cpt.70560. Online ahead of print. doi: 10.1002/cpt.70560 (PMC13755603; doi:10.1002/cpt.70560)
Supplement: Supplementary file 3 [file CPT-9999-0-s003.docx]

**Supplementary Text S2: Supplementary Results**

**Clinical study database**

From the CEPAM raw database, patients were excluded from the analysis due to various reasons (Figure 1):

(1) 575 patients were excluded due to not meeting the eligibility criteria for inclusion in the analysis: As the original model had been developed for female patients that had reached TAM steady state and to avoid biasing parameter estimates through potentially false assumptions regarding dosing history for patients not yet in TAM steady state, male patients (n=4) and patients not yet in TAM steady state (n=252) were excluded; patients with missing TAM (n=85) or ENDX (n=2) measurements or missing *CYP2D6* genotype (n=232) were excluded as this data was required for the analysis.
(2) Next, five studies were removed due to TAM or ENDX measurements deviating substantially and without explanatory factors from the rest of the database as previously described^1^: Study 4 (n=59) had substantially higher and study 14 (n=109) substantially lower TAM measurements; studies 16 and 27 (n=252) had substantially higher ENDX measurements; and study 13 (n=266) had a substantially higher TAM/ENDX ratio compared to the other studies.
(3) Finally, 93 patients were excluded due to implausible reported dosing or pharmacokinetic data: Patients with TAM (n=69) or ENDX (n=4) measurements reported as zero, with time after last dose reported as zero or >48h (n=18), one patient with a reported TAM dose of 1 mg QD and one patient with a reported TAM/ENDX ratio <0.1 were excluded from the analysis.

After exclusion of patients and studies, three studies (5, 21 and 28, n=269) showed a substantially lower total ENDX/TAM ratios than the other studies, while one study (17, n=173) showed substantially higher Z-ENDX/TAM ratios. In addition, all of these studies had reported insufficient details on the separation of ENDX isomers during bioanalysis. Hence, studies 5, 21 and 28 were re-assigned to Z-ENDX and study 17 was re-assigned to total ENDX as previously described^1^.

In the final analysis dataset, in addition to Z-ENDX or total ENDX, Z4’-ENDX measurements were available for 19.4% of patients, all from the Z-ENDX group. For some covariates, high amounts of missing data were observed (Table 1). Of note, the magnitude of missing data for time after last dose differed considerably between samples from patients with one vs. multiple sampling time points (Figure S1), with 81.5% vs. 0.801%, respectively.

**Development of *CYP2D6*-APA**

**Extension of PK NLME model.** During extension of the structural model, the previously unidentifiable parameters TAM absorption rate constant (k_a_), absorption lag time (t_lag_) and TAM volume of distribution (V_TAM,C_) were estimated with very good precision (≤16% RSE in final model). While the estimates for k_a_ and t_lag_ were close to the previously fixed values, the estimate for V_TAM,C_ decreased from 912 L to 770 L.

Following changes in the structural model, covariates on Z-ENDX formation, i.e., body weight, *CYP2D6* activity score, CYP2D6 inhibitor co-medication and *CYP2C9* genotype-predicted phenotype, were removed. In addition, non-adherence effect on TAM bioavailability was removed as it had been defined via TAM ≤60 ng/mL as surrogate^1^ and new data was available in the extended analysis dataset. In the final model, joint RUVs across patients with one vs. multiple sampling time points for TAM and Z-ENDX, respectively, increased IIV on Z-ENDX formation from 28.0% CV to 52.2% CV.

**Analysis of CYP2D6-independent influential factors**

The following covariates were tested for inclusion in the model based on observed trends in plots of covariate vs. ETA: CYP3A4 inducer co-medication on TAM clearance (CL_TAM_), *CYP2C9* and *CYP3A4* genotype-predicted phenotype on CYP2D6-independent Z-ENDX formation (CL_other_). The following covariates were tested for inclusion based on physiological plausibility or previous inclusion in the model: Non-adherence on TAM bioavailability (F), age and weight on volumes of distribution of TAM (V_TAM_), Z-ENDX (V_Z-ENDX_) and Z4’-ENDX (V_Z4’-ENDX_) and clearance of Z-ENDX (CL_Z-ENDX_) and Z4’-ENDX (CL_Z4’-ENDX_). After forward inclusion, the following covariates were newly included in the full model: Non-adherence on F, CYP3A4 inducer on CL_TAM_, *CYP2C9* genotype-predicted-phenotype on CL_other_, age on V_TAM_ and weight on CL_Z-ENDX_ and CL_Z4’-ENDX_. Upon backward deletion, the following covariates were removed from the model: age on V_TAM_ and CYP3A4 inducer co-medication on CL_TAM_.

During a sensitivity analysis to test the impact of missing covariate information, comparable parameter estimates were observed for covariate effects age, weight, non-adherence, CYP2D6 inhibitor co-medication, CYP2C9 and CYP3A4 phenotype.

**Assessment of impact of clinical influential factors on Z-ENDX**

Figure S4 illustrates theoretical covariate effects of CYP2D6-dependent and CYP2D6-independent covariates on minimal steady-state Z-ENDX concentrations at 24 hours after TAM administration: Apart from non-adherence, only no and reduced function *CYP2D6* alleles and strong CYP2D6 inhibitors reduced Z-ENDX concentrations significantly, i.e., 90% confidence intervals (CI) did not overlap with the reference exposure and were outside of the commonly used criteria for bioequivalence (0.8-1.25-fold of reference exposure). Worst-case settings combining multiple effects reduced minimal Z-ENDX concentration ≤33-fold, confirming the potential clinical relevance of impaired CYP2D6 activity and non-adherence as key covariates for predicting Z-ENDX concentration. A best-case setting including all covariate effects resulted in a weaker, though significant increase in minimal Z-ENDX concentration (1.8-fold).

**References**

1.	Mc Laughlin, A. M. *et al.* Nonlinear Mixed-Effects Model of Z-Endoxifen Concentrations in Tamoxifen-Treated Patients from the CEPAM Cohort. *Clin. Pharmacol. Ther.* **116**, 690–702 (2024).
