# Supplementary material for: A Precise Activity System for Pharmacogenetics Based on Estimation of cyp2d6 allelic Percentage Activity from the International CEPAM Database
Source: Clin Pharmacol Ther. 2026 Nov 11:10.1002/cpt.70560. Online ahead of print. doi: 10.1002/cpt.70560 (PMC13755603; doi:10.1002/cpt.70560)
Supplement: Supplementary file 4 [file CPT-9999-0-s004.docx]

**Supplementary Tables**

**Table S1.** Overview of studies included in the CEPAM database. Adapted from Mc Laughlin et al.^1^

|  | Study 1 | Study 2 | Study 3 | Study 4 | Study 5 |
| --- | --- | --- | --- | --- | --- |
| Study name/Title of publication | Pharmacology of adjuvant hormonotherapy in breast cancer (PHACS)^2,3^ | *CYP3A4*22* is related to increased plasma levels of 4-hydroxytamoxifen and partially compensates for reduced CYP2D6 activation of tamoxifen^4^ | Cytochrome P450 2D6 activity predicts discontinuation of tamoxifen therapy in breast cancer patients^5^ | Effects of CYP2D6 and UGT2B7 polymorphisms on pharmacokinetics of tamoxifen in Thai breast cancer patients^6^ | Dose-adjustment study of tamoxifen based on *CYP2D6* genotypes in Japanese breast cancer patients^7^ |
| Enrolled patients | 879 | 178 | 297 | 95 | 98 |
| Pharmacokinetic sampling | Trough sample every six months for 3 years (only one sample per patient available) | One sample 16-24h after last TAM intake | One sample per patient available | One sample per patient available | One sample per patient available |
| Matrix | Plasma | Plasma | Plasma | Plasma | Plasma |
| Sample handling after collection | **Liquid-liquid extraction**^8^   - Alkalinised with 1 M sodium hydroxide - Addition of dichlormethane/ isopropanol/hexane (1/1.3/2.5 v/v/v) - Organic phase dried at 37°C under air stream - Reconstitution in ACN/0.02 M ammonium formate buffer | **Liquid-liquid** **extraction**^4,9^   - Alkalinised with Tris buffer - Addition of hexane/n-propanol (95/5 v/v) - Organic phase dried under air stream - Reconsitution in mobile phase | **Liquid-liquid extraction**^10^   - Alkalinised with 1 M NaOH–glycine buffer - Addition of hexane/ isopropyl alcohol (95/5 v/v) - Organic phase dried under nitrogen - Reconstitution in acetonitrile and mobile phase (4:1 v/v) | **Protein precipitation**^6,11^   - With ACN - Exposure of supernatant to UV light (375 nm) for 20 min | **Protein precipitation**^7^   - With ACN |
| Analytical method | **UPLC-MS/MS**^8^   - Column: Acquity UPLC BEH C18, Temp. = 50°C - Mobile phase A: 0.1% formic acid in 2 mM ammonium formate aqueous solution - Mobile phase B: 0.1% formic acid in ACN | **UPLC-MS/MS**^9^   - Column: Acquity C18, Temp. = 50°C - Mobile phase A: 0.1% formic acid in aqueous solution - Mobile phase B: 0.1% formic acid in ACN | **HPLC with fluorescence detection**^10,12^   - Column: Regis Chemical C18 guard, Temp. NR - Mobile phase: 35% acetonitrile in 20 mM potassium phosphate buffer (pH 3) | **HPLC with fluorescence detector**^6,11^   - Column: Agilent Zorbax Extend-C18, Temp. = 35°C - Mobile phase: 1% triethanol-amin aqueous solution pH 11/methanol (18/82 v/v) - Fluorescence detector wavelength: excitation at 260 nm, emission at 375 nm | **UPLC-MS/MS**^7^   - Column: Acquity UPLC BEH C18, Temp. = 40°C - Mobile phase A: 10 mM ammonium formate aqueous solution - Mobile phase B: ACN |
| Endoxifen isomers identified | Z-endoxifen | Z-endoxifen | Total endoxifen | Total endoxifen | Total endoxifen |
| ACN: acetonitrile, HPLC: high-performance liquid chromatography, MS/MS: tandem mass spectrometry, NR: not reported, UPLC: ultra-performance liquid chromatography. | | | | | |

|  | Study 6 | Study 7 | Study 8 | Study 9 | Study 10 |
| --- | --- | --- | --- | --- | --- |
| Study name/Title of publication | Increasing tamoxifen dose in breast cancer patients based on *CYP2D6* genotypes and endoxifen levels: Effect on active metabolite isomers and the antiestrogenic activity score^13^ | Genotype-guided tamoxifen dosing increases active metabolite exposure in women with reduced CYP2D6 metabolism: A multicenter study^14^ | Comprehensive assessment of cytochromes P450 and transporter genetics with endoxifen concentration during tamoxifen treatment^15^ | Dose escalation of tamoxifen in patients with low endoxifen level: Evidence for Therapeutic Drug Monitoring - the TADE study^16^ | Relationship between SULT1A2 and CYP2D6 and tamoxifen metabolism in breast cancer patients^17^ |
| Enrolled patients | 121 | 119 | 500 | 122 | 135 |
| Pharmacokinetic sampling | 2-5 samples collected over 5 weeks (one sample per patient available) | One sample per patient available | One sample per patient available | Trough sample at least 8 weeks after last dosing change | Trough sample after 3-60 months of TAM treatment |
| Matrix | Plasma | Plasma | Plasma | Serum | Plasma |
| Sample handling after collection | **Protein precipitation**^18^   - With methanol - Supernatant diluted with water | **Liquid-liquid extraction**^14^   - Alkalinised - Addition of ethyl acetate - Organic phase dried - Reconstitution in mobile phase | **Liquid-liquid extraction**^14,19^   - Alkalinised - Addition of ethyl acetate - Organic phase dried - Reconstitution in mobile phase | **Protein precipitation**^20^   - With ACN - Dilution of supernatant with 3.5 mM ammonium formate buffer - Addition of ethyl acetate | **Protein precipitation**^17^   - With 1% formic acid in ACN - Supernatant applied to HybridSPE column |
| Analytical method | **HPLC-MS/MS**^18^   - Column: Zorbax SB-C18, Temp. = 55°C - Mobile phase A: 0.1% formic acid in methanol - Mobile phase B: 0.1% formic acid aqueous solution | **HPLC-MS/MS**^14^   - Column: Luna C18-2, Temp. NR - Mobile phase A: methanol/ 0.05% formic acid aqueous solution (45/55 v/v) - Mobile phase B: methanol/ 0.05% formic acid aqueous solution (90:10 v/v) | **HPLC-MS/MS**^14^   - Column: Luna C18-2, Temp. NR - Mobile phase A: methanol/ 0.05% formic acid aqueous solution (45/55 v/v) - Mobile phase B: methanol/ 0.05% formic acid aqueous solution (90:10 v/v) | **HPLC-MS/MS**^16^   - Column: Synergi Hydro-RP, Temp. = 40°C - Mobile phase A: water - Mobile phase B: ACN - Mobile phase C: 34 mM ammonium formate aqueous solution | **HPLC-MS/MS**^17^   - Column: Zorbax Eclipse XDB-C18, Temp. = 30°C - Mobile phase A: 0.1% formic acid aqueous solution - Mobile phase B: 0.1% formic acid in ACN |
| Endoxifen isomers identified | Z-endoxifen | Total endoxifen | Z-endoxifen | Z-endoxifen | Total endoxifen |
| ACN: acetonitrile, HPLC: high-performance liquid chromatography, MS/MS: tandem mass spectrometry, NR: not reported, UPLC: ultra-performance liquid chromatography. | | | | | |

|  | Study 11 | Study 12 | Study 13 | Study 14 | Study 15 |
| --- | --- | --- | --- | --- | --- |
| Study name/Title of publication | Variations in plasma concentrations of tamoxifen metabolites and the effects of genetic polymorphisms on tamoxifen metabolism in Korean patients with breast cancer^21^ | *CYP2D6* genotypes, endoxifen levels, and disease recurrence in 224 Filipino and Vietnamese women receiving adjuvant tamoxifen for operable breast cancer^22^ | Limited predictive value of achieving beneficial plasma (Z)-endoxifen threshold level by *CYP2D6* genotyping in tamoxifen-treated Polish women with breast cancer^23^ | Multigene and drug interaction approach for tamoxifen metabolite patterns reveals possible involvement of CYP2C9, CYP2C19, and ABCB1^24^ | Serum concentrations of active tamoxifen metabolites predict long-term survival in adjuvantly treated breast cancer patients^25^ |
| Enrolled patients | 281 | 224 | 285 | 116 | 99 |
| Pharmacokinetic sampling | Trough sample at 8 weeks, 3 months and later (one sample per patient available) | One sample at four or more months of TAM treatment | One sample at 1-70 months of TAM treatment | One sample within 24h after last TAM dose | One sample 3 years after inclusion |
| Matrix | Plasma | Plasma | Plasma | Plasma | Serum |
| Sample handling after collection | **Protein precipitation**^21^   - With ACN | **Liquid-liquid extraction**^14^   - Alkalinised - Addition of ethyl acetate - Organic phase dried - Reconstitution in mobile phase | **Protein precipitation**^23^   - With ACN - Organic phase dried under nitrogen at 55°C - Reconstitution in 10 mM ammonium formate in methanol/water (1/1 v/v) | **Protein precipitation**^24^   - With ACN/methanol (1/1 v/v) | **Protein precipitation**^25^   - With ACN - Organic phase dried under nitrogen - Reconstitution in methanol - Dilution in water/methanol (20/80 v/v) |
| Analytical method | **HPLC-MS/MS**^21^   - Column: Poroshell 120 EC-C18, Temp. NR - Mobile phase A: 0.1% formic acid in 2 mM ammonium acetate aqueous solution - Mobile phase B: 0.1% formic acid in 2 mM ACN aqueous solution | **HPLC-MS/MS**^14^   - Column: Luna C18-2, Temp. NR - Mobile phase A: methanol/ 0.05% formic acid aqueous solution (45/55 v/v) - Mobile phase B: methanol/ 0.05% formic acid aqueous solution (90/10 v/v) | **UPLC-MS**/MS^23^   - Column: Acquity BEH Shield RP18, Temp. = 70°C - Mobile phase A: water - Mobile phase B: 0.1% formic acid in ACN | **UPLC-MS/MS**^24^   - Column: Acquity UPLC Charged Surface Hybrid C18, Temp. = 50°C - Mobile phase A: 0.1% formic acid in water - Mobile phase B: 0.1% formic acid in ACN | **UPLC-MS/MS**^25^   - Column: BEH Phenyl, Temp. = 50°C - Mobile phase A: 0.01% formic acid aqueous solution - Mobile phase B: 0.01% formic acid in methanol |
| Endoxifen isomers identified | Total endoxifen | Total endoxifen | Z-endoxifen | Total endoxifen | Z-endoxifen |
| ACN: acetonitrile, HPLC: high-performance liquid chromatography, MS/MS: tandem mass spectrometry, NR: not reported, UPLC: ultra-performance liquid chromatography. | | | | | |

|  | Study 16 | Study 17 | Study 18 | Study 19 | Study 20 |
| --- | --- | --- | --- | --- | --- |
| Study name/Title of publication | Effects of *CYP2D6* and *SULT1A1* genotypes including *SULT1A1* gene copy number on tamoxifen metabolism^26^ | Clinical implications of *CYP2D6* genotypes predictive of tamoxifen pharmacokinetics in metastatic breast cancer^27^ | Activity levels of tamoxifen metabolites at the estrogen receptor and the impact of genetic polymorphisms of phase I and II enzymes on their concentration levels in plasma^28^ | Tamoxifen metabolism predicts drug concentrations and outcome in premenopausal patients with early breast cancer^29^ | Tamoxifen pharmacogenetics and metabolism: Results from the prospective CYPTAM Study^30^ |
| Enrolled patients | 151 | 212 | 245 | 587 | 667 |
| Pharmacokinetic sampling | One trough sample per patient available | One sample after at least 8 weeks of TAM treatment | Sample at month 6, 12 of TAM treatment (one sample per patient available) | Steady-state sample within first year of treatment | Trough steady-state sample |
| Matrix | Serum | Plasma | Plasma | Plasma | Serum |
| Sample handling after collection | **Protein precipitation**^31^   - With ACN | **Liquid-liquid extraction**^10^   - Alkalinised with 1 M sodium hydroxide glycine buffer - Addition of hexane/ isopropyl alcohol (95/5 v/v) - Organic phase dried under nitrogen - Reconstitution in ACN/mobile phase (4/1 v/v) | **Protein precipitation**^28^   - With 1% acetic acid in ACN - Dilution of supernatant with 0.1% acetic acid aqueous solution | **Protein preciptitation**^28^   - With 1% acetic acid in ACN - Dilution of supernatant with 0.1% acetic acid aqueous solution | **Protein precipitation**^20^   - With ACN - Dilution of supernatant with 3.5 mM ammonium formate buffer |
| Analytical method | **HPLC-MS/MS**^31^   - Column: Chromolith Performance RP-18e, Temp. NR - Mobile phase A: water - Mobile phase B: 0.05% formic acid in water - Mobile phase C: ACN | **HPLC with fluorescence detector**^10,27^   - Column: Capcell Pak C18, Temp. NR - Mobile phase: ACN/20 mM potassium phosphate buffer (35/65 v/v) - Eluents pass PHRED photochemical reactor - Fluorescence detector wavelength: excitation at 256 nm, emission at 380 nm | **HPLC-MS/MS**^28^   - Column: ZORBAX Eclipse plus C18, Temp. = 45°C - Mobile phase A: ACN - Mobile phase B: 0.1% acetic acid aqueous solution | **HPLC-MS/MS**^28^   - Column: ZORBAX Eclipse plus C18, Temp. = 45°C - Mobile phase A: ACN - Mobile phase B: 0.1% acetic acid aqueous solution | **HPLC-MS/MS**^20,30^   - Column: Synergi Hydro column, Temp. = 40 °C - Mobile phase A: 98% formic acid in 3.5 mM ammonium formate solution (pH 3.5) - Mobile phase B: ACN |
| Endoxifen isomers identified | Total endoxifen | Z-endoxifen | Z-endoxifen | Z-endoxifen | Total endoxifen |
| ACN: acetonitrile, HPLC: high-performance liquid chromatography, MS/MS: tandem mass spectrometry, NR: not reported, UPLC: ultra-performance liquid chromatography. | | | | | |

|  | Study 21 | Study 22 | Study 23 | Study 24 | Study 25 |
| --- | --- | --- | --- | --- | --- |
| Study name/Title of publication | Hormonal status affects plasma exposure of tamoxifen and its main metabolites in tamoxifen-treated breast cancer patients^32^ | Evaluation of CYP2D6 enzyme activity using a dextromethorphan breath test in women receiving adjuvant tamoxifen^33^ | Drug monitoring of tamoxifen metabolites predicts vaginal dryness and verifies a low discontinuation rate from the Norwegian Prescription Database^34^ | Tamoxifen metabolite concentrations, *CYP2D6* genotype, and breast cancer outcomes^35^ | Association of *CYP2D6* genotype and tamoxifen metabolites with breast cancer recurrence in a low-dose trial^36^ |
| Enrolled patients | 40 | 92 | 220 | 1370 | 154 |
| Pharmacokinetic sampling | Dense sampling at 0, 0.5, 1, 1.5, 2, 3, 4, 6, 8, 12, 16, 24h after at least 3 months (one sample per patient available) | One sample after at least 6 months on TAM treatment | One sample every 6 or 12 months (one sample per patient available) | One sample per patient available | One sample at baseline, year 1, 3 (one sample per patient available) |
| Matrix | Plasma | Plasma | Serum | Serum | Serum |
| Sample handling after collection | **Liquid-liquid extraction**^32^   - Alkalinised with 1 M sodium hydroxide - Addition of methyl tert-butyl ether | **Liquid-liquid extraction**^28,31^   - Alkalinised with 1 M sodium hydroxide glycine buffer - Addition of hexane/ isopropyl alcohol (95/5 v/v) - Organic phase dried - Reconstitution in mobile phase | **Protein precipitation**^25,34^   - With ACN - Organic phase dried under nitrogen - Reconstitution in water/methanol (1/1 v/v) | **Solid-phase extraction**^35^   - 0.5 mM ammonium formate buffer - Analyte extraction using Waters MCX 1cc solid-phase extraction cartridges | **Protein precipitation**^25,36^   - With ACN - Organic phase dried under nitrogen - Reconstitution in water/methanol (20/80 v/v) |
| Analytical method | **HPLC-MS/MS**^32^   - Column: RP-Select B LiChroCART C18, Temp. NR - Mobile phase: 0.1% formic acid in 10 mM ammonium formate/ACN (1/1 v/v) | **HPLC with fluorescence detection**^28,31^   - Column: C18 reverse phase, Temp. NR - Mobile phase: ACN/20 mM potassium phosphate buffer (35/65 v/v) - Eluents pass PHRED photochemical reactor - Fluorescence detector wavelength: excitation at 250 nm, emission at 370 nm | **UPLC-MS/MS**^25,34^   - Column: BEH Phenyl, Temp. = 50°C - Mobile phase A: 0.01% formic acid - Mobile phase B: 0.01% formic acid in methanol | **UPLC-MS/MS**^35^   - Column: Waters XTerra MS C18, Temp. NR - Mobile phase: NR | **UPLC-MS/MS**^25,36^   - Column: Waters BEH Phenyl, Temp. = 50°C - Mobile phase A: 0.01% formic acid aqueous solution - Mobile phase B: 0.01% formic acid in methanol |
| Endoxifen isomers identified | Total endoxifen | Z-endoxifen | Z-endoxifen | Z-endoxifen | Z-endoxifen |
| ACN: acetonitrile, HPLC: high-performance liquid chromatography, MS/MS: tandem mass spectrometry, NR: not reported, UPLC: ultra-performance liquid chromatography. | | | | | |

|  | Study 26 | Study 27 | Study 28 | Study 29 | Study 30^a^ |
| --- | --- | --- | --- | --- | --- |
| Study name/Title of publication | Impairment of endoxifen formation in tamoxifen-treated premenopausal breast cancer patients carrying reduced- function *CYP2D6* alleles^37^ | Randomized double-blind 2 x 2 trial of low-dose tamoxifen and fenretinide for breast cancer prevention  in high-risk premenopausal women^38^ | Endoxifen levels and metabolic  phenotype-associated factors in Mexican Mestizo patients under tamoxifen treatment^39^ | Impact of curcumin (with or without piperine) on the pharmacokinetics of tamoxifen^40^ | Influence of green tea consumption on endoxifen steady‐state concentration in breast cancer patients treated with tamoxifen^41^ |
| Enrolled patients | 118 | 235 | 138 | 16 | 14 |
| Pharmacokinetic sampling | One sample per patient available | One sample every six months (one sample per patient available) | One sample per patient available | Dense sampling at 0, 0.5, 1, 1.5, 2, 2.5, 3, 3.5, 4, 6, 8, 12, 24h at days 28, 56, 84 | Dense sampling at 0, 0.5, 1, 1.5, 2, 2.5, 3, 3.5, 4, 6, 8, 12, 24h at days 14, 42 |
| Matrix | Plasma | Serum | Plasma | Plasma | Plasma |
| Sample handling after collection | **Protein precipitation**^37^   - With 0.08 M zinc sulphate in methanol/water (80/20) - Supernatant dried under nitrogen or vacuum centrifugation - Reconstitution in methanol | **Protein precipitation**^31^   - With ACN | **Analyte extraction**^42^   - Methanol - Alkalinised with potassium phosphate buffer - Hexane/isobutanol (68/32 v/v) - Organic phase dried - Reconstitution in mobile phase with 10% methanol | **Liquid-liquid extraction**^43^   - Alkalinised with glycine buffer (pH 11.5) - Addition of hexane/ isopropyl alcohol (95/5 v/v) - Organic phase dried under nitrogen - Reconstitution in ACN/ water/formic acid (40:60:0.1 v/v/v) | Details NR |
| Analytical method | **UPLC-MS/MS**^37^   - Column: Accucore C18, Temp. NR - Mobile phase A: 0.005% formic acid in 10 mM ammonium formate aqueous solution - Mobile phase B: 0.005% formic acid in methanol | **HPLC-MS/MS**^31^   - Column: Chromolith performance RP-18e, Temp. NR - Mobile phase A: water - Mobile phase B: 0.05% formic acid in water - Mobile phase C: ACN | **HPLC with fluorescence detection**^42^   - Column: Zorbax Eclipse plus C18, Temp. = 57°C - Mobile phase A: 35 nM potassium phosphate aqueous solution/ACN (38/62 v/v) - Mobile phase B: ACN - Fluorescence detector wavelength: excitation at 260 nm, emission at 375 nm | **UPLC-MS/MS**^43^   - Column: Acquity UPLC BEH C18, Temp. = 50°C - Mobile phase A: 0.1% formic acid in 0.2mM ammonium formate aqueous solution - Mobile phase B: 0.1% formic acid in ACN | **UPLC-MS/MS**^41^   - Details NR |
| Endoxifen isomers identified | Total endoxifen | Z-endoxifen | Z-endoxifen | Z-endoxifen | Z-endoxifen |
| ACN: acetonitrile, HPLC: high-performance liquid chromatography, MS/MS: tandem mass spectrometry, NR: not reported, UPLC: ultra-performance liquid chromatography.  ^a^Some patients were enrolled in several of studies 30-34. | | | | | |

|  | Study 31^a^ | Study 32^a^ | Study 33^a^ | Study 34^a^ | Study 35 |
| --- | --- | --- | --- | --- | --- |
| Study name/Title of publication | Influence of probenecid on endoxifen systemic exposure in breast cancer patients on adjuvant tamoxifen treatment^44^ | CBD-oil as a potential solution in case of severe tamoxifen-related side effects^45^ | TDM of tamoxifen (TOTAM)^46,47^ | The impact of endoxifen-guided tamoxifen dose reductions on endocrine side-effects in patients with primary breast cancer (TOTAM)^48^ | (Z)-Endoxifen and early recurrence of breast cancer: An explorative analysis in a prospective Brazilian study^49^ |
| Enrolled patients | 11 | 15 | 303 | 149 | 149 |
| Pharmacokinetic sampling | Dense sampling at 14 timepoints over 24h at days 7, 21 | Dense sampling at 0, 0.5, 1, 1.5, 2, 2.5, 3.5, 4, 6, 8, 10, 24h at days 7, 35 | Trough sample at months 3, 4.5, 6 | Trough sample at months 3, 4.5, 6 or 6 weeks, 3 months after dose reduction | One sample at months 3, 6, 12 |
| Matrix | Plasma | Plasma | Plasma | Plasma | Plasma |
| Sample handling after collection | **Liquid-liquid extraction**^43^   - Alkalinised with glycine buffer (pH 11.5) - Addition of hexane/ isopropyl alcohol (95/5 v/v) - Organic phase dried under nitrogen - Reconstitution in ACN/ water/formic acid (40:60:0.1 v/v/v) | **Liquid-liquid extraction**^43^   - Alkalinised with glycine buffer (pH 11.5) - Addition of hexane/ isopropyl alcohol (95/5 v/v) - Organic phase dried under nitrogen - Reconstitution in ACN/ water/formic acid (40:60:0.1 v/v/v) | **Liquid-liquid extraction**^43^   - Alkalinised with glycine buffer (pH 11.5) - Addition of hexane/ isopropyl alcohol (95/5 v/v) - Organic phase dried under nitrogen - Reconstitution in ACN/ water/formic acid (40:60:0.1 v/v/v) | **Liquid-liquid extraction**^43^   - Alkalinised with glycine buffer (pH 11.5) - Addition of hexane/ isopropyl alcohol (95/5 v/v) - Organic phase dried under nitrogen - Reconstitution in ACN/ water/formic acid (40:60:0.1 v/v/v) | **Protein precipitation**^28^   - With 1% acetic acid in ACN - Dilution of supernatant with 0.1% acetic acid aqueous solution |
| Analytical method | **UPLC-MS/MS**^43^   - Column: Acquity UPLC BEH C18, Temp. = 50°C - Mobile phase A: 0.1% formic acid in 0.2mM ammonium formate aqueous solution - Mobile phase B: 0.1% formic acid in ACN | **UPLC-MS/MS**^43^   - Column: Acquity UPLC BEH C18, Temp. = 50°C - Mobile phase A: 0.1% formic acid in 0.2mM ammonium formate aqueous solution - Mobile phase B: 0.1% formic acid in ACN | **UPLC-MS/MS**^43^   - Column: Acquity UPLC BEH C18, Temp. = 50°C - Mobile phase A: 0.1% formic acid in 0.2mM ammonium formate aqueous solution - Mobile phase B: 0.1% formic acid in ACN | **UPLC-MS/MS**^43^   - Column: Acquity UPLC BEH C18, Temp. = 50°C - Mobile phase A: 0.1% formic acid in 0.2mM ammonium formate aqueous solution - Mobile phase B: 0.1% formic acid in ACN | **HPLC-MS/MS**^28^   - Column: ZORBAX Eclipse plus C18, Temp. = 45°C - Mobile phase A: ACN - Mobile phase B: 0.1% acetic acid aqueous solution |
| Endoxifen isomers identified | Z-endoxifen | Z-endoxifen | Z-endoxifen | Z-endoxifen | Z-endoxifen |
| ACN: acetonitrile, HPLC: high-performance liquid chromatography, MS/MS: tandem mass spectrometry, NR: not reported, UPLC: ultra-performance liquid chromatography.   ^a^Some patients were enrolled in several of studies 30-34. | | | | | |

|  | Study 36 |  |
| --- | --- | --- |
| Study name/Title of publication | Effects of genetic polymorphisms of drug metabolizing enzymes and co-medications on tamoxifen metabolism in Black South African women with breast cancer^50^ |  |
| Enrolled patients | 229 |  |
| Pharmacokinetic sampling | One sample 12-15h since last TAM dose |  |
| Matrix | Plasma |  |
| Sample handling after collection | **Protein precipitation**^50^   - With ACN - Supernatant dried under nitrogen - Reconstitution in mobile phase |  |
| Analytical method | **HPLC-MS/MS**^50^   - Column: Zorbax C18,  Temp. = 40°C - Mobile phase A: 0.1% formic acid in 10 mM ammonium formate solution - Mobile phase B: 0.1% formic acid in ACN |  |
| Endoxifen isomers identified | Total endoxifen |  |
| ACN: acetonitrile, HPLC: high-performance liquid chromatography, MS/MS: tandem mass spectrometry, NR: not reported, UPLC: ultra-performance liquid chromatography. | | |

**Table S2.** Overview of *CYP2D6* genotyping assays used in the CEPAM database. Adapted from Mc Laughlin et al.^1^

|  | *CYP2D6* genotyping assay | *CYP2D6* alleles determined | *CYP2D6* Copy Number Variation tested | | Missing alleles compared to Roche AmpliChip | *CYP3A4* alleles determined | *CYP2C9* alleles determined |
| --- | --- | --- | --- | --- | --- | --- | --- |
|  |  |  | **Deletion** | **Duplication** |  |  |  |
| Study 1 | Biomark (Fluidigm) in a microfluidic multiplex 96.96 dynamic array chip^2^ | **4, *5, *6, *7, *9, *10, *17, *41* | Y | Y | **2, *3, *8, *11, *14, *15, *19, *20, *25, *26, *29, *30, *31, *35, *36, *40* | **1B, *22* | - |
| Study 2 | Luminex xTAG® *CYP2D6* Kit v3^4^ | **2, *3, * 4, *5, *6, *7, *8, *9, *10, *11, *15, *17, *29, *35, *41* | Y | Y | **14, *19, *20, *25, *26, *30, *31, *36, *40* | **22* | - |
| Study 3 | Roche AmpliChip^5,12^ | **2, *3, *4, *5, *6, *7, *8, *9, *10, *11, *14A, *14B, *15, *17, *19, *20, *25, *26, *29, *30, *31, *35, *36, *40, *41* | Y | Y | - | - | - |
| Study 4 | TaqMan® Drug Metabolism Genotyping Assay Mix^6^ | **10* | - | - | **2, *3, *4, *5, *6, *7, *8, *9, *11, *14, *15, *17, *19, *20, *25, *26, *29, *30, *31, *35, *36, *40, *41* | - | - |
| Study 5 | Real-time Invader (Third Wave Technologies, Madison,WI, USA), TaqMan assays^7,51^ | **4, *5, *6, *10, *14, *18, *21, *36, *41* | Y | Y | **2, *3, *7, *8, *9, *11, *15, *17, *19, *20, *25, *26, *29, *30, *31, *35, *40* | - | - |
| Study 6 | Tag-It™ Mutation Detection Kits (Tm Bioscience, Toronto, ON, Canada)^13,52^ | **2A, *2, *3, *4, *5, *6, *7, *8, *9, *10, *11, *12, *17* | Y | Y | **14, *15, *19, *20, *25, *26, *29, *30, *31, *35, *36, *40, *41* | - | - |
| Study 7 | Roche AmpliChip^14^ | **2, *3, *4, *5, *6, *7, *8, *9, *10, *11, *14A, *14B, *15, *17, *19, *20, *25, *26, *29, *30, *31, *35, *36, *40, *41* | Y | Y | - | - | - |
| Study 8 | Roche AmpliChip^15^ | **2, *3, *4, *5, *6, *7, *8, *9, *10, *11, *14A, *14B, *15, *17, *19, *20, *25, *26, *29, *30, *31, *35, *36, *40, *41* | Y | Y | - | **20, *22* | **2, *3, *5, *6, *8, *11, *12* |
| Study 9 | Single base extension assay (iplex) analyzed on a Sequenom Massarray MALDI-TOF mass spectrometer (Sequenom)^16^ | **2, *3, *4, *5, *6, *7, *8, *9, *10, *14, *14B, *18, *41* | Y | Y | **11, *15, *17, *19, *20, *25, *26, *29, *30, *31, *35, *36, *40* | - | - |
| Study 10 | Roche AmpliChip^17^ | **2, *3, *4, *5, *6, *7, *8, *9, *10, *11, *14A, *14B, *15, *17, *19, *20, *25, *26, *29, *30, *31, *35, *36, *40, *41* | Y | Y | - | **1B, *3, *17* | - |
|  | ***CYP2D6* genotyping assay** | ***CYP2D6* alleles determined** | ***CYP2D6* Copy Number Variation tested** | | **Missing alleles compared to Roche AmpliChip** | ***CYP3A4* alleles determined** | ***CYP2C9* alleles determined** |
|  |  |  | **Deletion** | **Duplication** |  |  |  |
| Study 11 | DMET Plus array (Affymetrix, Santa Clara, CA, USA)^21^ | **2, *5, *6, *10, *39, *41* | Y | Y | **3, *4, *7, *8, *9, *11, *14, *15, *17, *19, *20, *25, *26, *29, *30, *31, *35, *36, *40* | **5, *18* | **3, *7* |
| Study 12 | TaqMan Genotyping Assays (Applied Biosystems, Foster City, CA, USA)^22^ | **2, *3, *4, *5, *6, *10, *41* | Y | - | **7, *8, *9, *11, *14, *15, *17, *19, *20, *25, *26, *29, *30, *31, *35, *36, *40* | - | - |
| Study 13 | TaqMan allelic discrimination assays^23^ | **2, *3, *4, *5, *6, *7, *9, *10, *17, *41* | Y | Y | **8, *11, *14, *15, *19, *20, *25, *26, *29, *30, *31, *35, *36, *40* | - | - |
| Study 14 | TaqMan-based genotype detection^24^ | **2, *3, *4, *5, *6, *7, *8, *9, *10, *11, *12, *14, *17, *29, *35, *41A* | Y | Y | **15, *19, *20, *25, *26, *30, *31, *36, *40* | **1B* | **2, *3A, *4, *5, *6, *8, *11* |
| Study 15 | INFINITI® CYP450 2D6I Assay, verified with TaqMan DME assay^25^ | **2, *2A, *3, *4, *5, *6, *7, *8, *9, *10, *12, *14, *17, *29, *41* | Y | Y | **11, *15, *19, *20, *25, *26, *30, *31, *35, *36, *40* | - | - |
| Study 16 | PCR and TaqMan probes with ABI Prism 7700 sequence detection system (Applied Biosystems)^26^ | **3, *4, *5, *6* | Y | Y | **2, *7, *8, *9, *10, *11, *14, *15, *17, *19, *20, *25, *26, *29, *30, *31, *35, *36, *40, *41* | - | - |
| Study 17 | PCR and digestion assay^27^ | **5, *10* | Y | Y | **2, *3, *4, *6, *7, *8, *9, *11, *14, *15, *17, *19, *20, *25, *26, *29, *30, *31, *35, *36, *40, *41* | - | - |
| Study 18 | TaqMan allelic discrimination assays and *CYP2D6* TaqMan Gene Copy Number Assay^28^ | **3, *4, *5, *6, *7, *8, *9, *10, *41* | Y | Y | **2, *11, *14, *15, *17, *19, *20, *25, *26, *29, *30, *31, *35, *36, *40* | - | **2, *3* |
| Study 19 | Matrix-assisted laser desorption/ ionization MS verified by Roche AmpliChip and TaqMan Copy Number assay^29^ | **3, *4, *5, *6, *9, *10, *14, *15, *17, *41* | Y | Y | **2, *7, *8, *11, *19, *20, *25, *26, *29, *30, *31, *35, *36, *40* | - | **2, *3* |
| Study 20 | Roche AmpliChip^30^ | **2, *3, *4, *5, *6, *7, *8, *9, *10, *11, *14A, *14B, *15, *17, *19, *20, *25, *26, *29, *30, *31, *35, *36, *40, *41* | Y | Y | - | - | - |
| Study 21 | Real-time PCR and TaqMan Copy Number assay^32^ | **2, *4, *5, *9, *10, *17, *29, *39, *41* | Y | Y | **3, *6, *7, *8, *11, *14, *15, *19, *20, *25, *26, *30, *31, *35, *36, *40* | **1B, *22* | - |
| Study 22 | Luminex platform verified by TaqMan assay or Sanger sequencing^33^ | **2, *2A, *3, *4, *5, *6, *7, *8, *9, *10, *11, *12, *17, *41* | Y | Y | **14, *15, *19, *20, *25, *26, *29, *30, *31, *35, *36, *40* | - | - |
|  | ***CYP2D6* genotyping assay** | ***CYP2D6* alleles determined** | ***CYP2D6* Copy Number Variation tested** | | **Missing alleles compared to Roche AmpliChip** | ***CYP3A4* alleles determined** | ***CYP2C9* alleles determined** |
|  |  |  | **Deletion** | **Duplication** |  |  |  |
| Study 23 | VeriDose® Core and VeriDose® *CYP2D6* CNV panels (Agenda Bioscience, San Diego, CA, USA)^53^ | **2, *3, *4, *5, *6*, *7, *8, *9, *10, *11, *12, *14*, *15, *17, *18, *19, *20, *29, *41* | Y | Y | **25, *26, *30, *31, *35, *36, *40* | **20, *22* | **2, *3, *5, *6, *8, *9, *11, *12,* |
| Study 24 | Roche AmpliChip^35^ | **2, *3, *4, *5, *6, *7, *8, *9, *10, *11, *14A, *14B, *15, *17, *19, *20, *25, *26, *29, *30, *31, *35, *36, *40, *41* | Y | Y | - | - | - |
| Study 25 | INFINITI CYP450 2DI assay^36^ | **2, *2A, *3, *4, *5, *6, *7, *8, *9, *10, *12, *14, *17, *29, *41* | Y | Y | **11, *15, *19, *20, *25, *26, *30, *31, *35, *36, *40* | - | - |
| Study 26 | TaqMan-based real-time PCR^37^ | **3, *4, *5, *6, *9, *10, *41* | Y | Y | **2, *7, *8, *11, *14, *15, *17, *19, *20, *25, *26, *29, *30, *31, *35, *36, *40* | - | - |
| Study 27 | INFINITI® CYP450 2D6I Assay^54^ | **2, *2A, *3, *4, *5, *6, *7, *8, *9, *10, *12, *14, *17, *29, *41* | Y | Y | **11, *15, *19, *20, *25, *26, *30, *31, *35, *36, *40* | - | - |
| Study 28 | TaqMan® Allelic Discrimination Assay (Applied Biosystems, CA, USA)^39^ | **2, *3, *4, *5, *10, *17, *29, *34, *39* | Y | Y | **6, *7, *8, *9, *11, *14, *15, *19, *20, *25, *26, *30, *31, *35, *36, *40, *41* | - | - |
| Study 29 | Quantstudio and Infiniti^40,46,47^ | **2, *3, *4, *5, *6, *7, *8, *9, *10, *12, *14, *17, *29, *41* | Y | Y | **11, *15, *19, *20, *25, *26, *30, *31, *35, *36, *40* | **22* | - |
| Study 30 | Quantstudio and Infiniti^41,46,47^ | **2, *3, *4, *5, *6, *7, *8, *9, *10, *12, *14, *17, *29, *41* | Y | Y | **11, *15, *19, *20, *25, *26, *30, *31, *35, *36, *40* | **22* |  |
| Study 31 | Quantstudio and Infiniti^44,46,47^ | **2, *3, *4, *5, *6, *7, *8, *9, *10, *12, *14, *17, *29, *41* | Y | Y | **11, *15, *19, *20, *25, *26, *30, *31, *35, *36, *40* | **22* | - |
| Study 32 | Quantstudio and Infiniti^45–47^ | **2, *3, *4, *5, *6, *7, *8, *9, *10, *12, *14, *17, *29, *41* | Y | Y | **11, *15, *19, *20, *25, *26, *30, *31, *35, *36, *40* | **22* | - |
| Study 33 | Quantstudio and Infiniti^46,47^ | **2, *3, *4, *5, *6, *7, *8, *9, *10, *12, *14, *17, *29, *41* | Y | Y | **11, *15, *19, *20, *25, *26, *30, *31, *35, *36, *40* | **22* | - |
| Study 34 | Quantstudio and Infiniti^46–48^ | **2, *3, *4, *5, *6, *7, *8, *9, *10, *12, *14, *17, *29, *41* | Y | Y | **11, *15, *19, *20, *25, *26, *30, *31, *35, *36, *40* | **22* | - |
| Study 35 | Matrix-assisted laser desorption/ ionization MS and TaqMan real-time quantification^49,55^ | **3, *4, *5, *6, *7, *8, *9, *10, *17, *29, *41* | Y | Y | **2, *11, *14, *15, *19, *20, *25, *26, *30, *31, *35, *36, *40* | - | - |
| Study 36 | GenoPharm open array panel with TaqMan-based quantification^50^ | **2, *4, *5, *6, *7, *10, *12, *17, *29, *41* | Y | Y | **3, *8, *9, *11, *14, *15, *19, *20, *25, *26, *30, *31, *35, *36, *40* | - | - |
| MS: mass spectrometry, PCR: polymerase chain reaction | | | | | | | |

**Table S3.** Frequency of *CYP2D6* alleles for patients in the CEPAM database genotyped with Roche AmpliChip. Allele frequencies were stratified by biogeographical group for groups with >100 patients.

|  | Overall | White | Asian | Black/African American |
| --- | --- | --- | --- | --- |
| Number of patients | 2774 | 2456 | 177 | 114 |
| *CYP2D6* allele frequency, % |  |  |  |  |
| **1* | 40.7 | 39.9 | 56.8 | 46.5 |
| **2* | 16.3 | 17.3 | 10.2 | 6.14 |
| **3* | 1.42 | 1.38 | 0.565 | 0 |
| **4* | 18.5 | 19.7 | 9.04 | 7.89 |
| **5* | 3.77 | 3.56 | 3.95 | 6.58 |
| **6* | 1.01 | 1.06 | 0.282 | 0 |
| **7*^a^ | 0.595 | 0.217 | 0 | 11.0 |
| **9* | 2.61 | 2.44 | 1.13 | 5.70 |
| **10* | 2.38 | 1.47 | 13.8 | 2.19 |
| **11* | 0.0180 | 0.0271 | 0 | 0 |
| **14* | 0.0180 | 0 | 0 | 0 |
| **15* | 0.0180 | 0.0271 | 0 | 0 |
| **17* | 0.523 | 0.244 | 0.282 | 7.02 |
| **19* | 0.0180 | 0.0271 | 0 | 0 |
| **29* | 0.126 | 0 | 0 | 2.19 |
| **35* | 4.22 | 4.42 | 0.847 | 0 |
| **36* | 0.0180 | 0.0271 | 0 | 0 |
| **40* | 0.0360 | 0 | 0 | 0.877 |
| **41* | 7.71 | 8.22 | 3.11 | 3.95 |
| CNV (Multiplication) | 2.07 | 2.25 | 0.565 | 4.82 |
| CNV: Copy number variations  ^a^Observed allele frequencies for *CYP2D6*7* deviate from frequencies expected based on gnomAD v4.1, especially for White (0.0388%) and Black/African American (0.00538%) patients.^56,57^ | | | | |

**Table S4.** *CYP2D6* allelic percentage activities (*CYP2D6*-APA, %) across evaluations for multiple substrates based on clinical pharmacokinetic data. Reported relative activities compared to *CYP2D6*1* were transformed to percent and confidence intervals were reported if they were available for relative activities.

|  | Current analysis | Van der Lee^58^ | Agema^59^ | Hertz^19^ | Haslemo^60^ | Jukić^61^ | Jukić^61^ | Jukić^61^ | Størset^62^ | Kehinde^63^ | Bennett^64^ | Frederik-sen^65^ | Frederik-sen^66^ | Frederik-sen^67^ | Abdulja-lil^68^ |
| --- | --- | --- | --- | --- | --- | --- | --- | --- | --- | --- | --- | --- | --- | --- | --- |
| Substrate | TAM | TAM | TAM | TAM^a^ | Venla-faxine^b,c^ | Venla-faxine^b,c^ | Aripipra-zole^c^ | Rispe-ridone^b,c^ | Rispe-ridone^b,d^ | Rispe-ridone^a^ | Rispe-ridone | Brexpi-prazole | Vortio-xetine^e^ | Tedatio-xetine^e^ | Dextrome-torphan |
| Patient number | 7097 | 561 | 539 | 355 | 1003 | 1742 | 1199 | 985 | 525 | 208 | 161 | 496 | 1140 | 578 | 36 |
| Method | PopPK | ML | PopPK | LR | LR | LR | LR | LR | PopPK | LR | LR | PopPK + LR | PopPK | PopPK + LR | PopPK |
| *CYP2D6*-*A*PA [95% CI], % | | | | | | | | | | | | | | | |
| **2* | 64 [60-68] | ~75 [-] | 56 [48-64] | 63 [-] | - | - | - | - | 66 [50-85] | 76 [-] | 60 [-] | 10 [5-15] | - | - | 40 [-] |
| **9* | 52 [44-61] | ~65 [-] | 38 [27-49] | 85 [-] | 34 [-] | 39 [31-48] | 25 [13-40] | 31 [15-51] | 40 [20-68] | - | 230^f^ [-] | 23 [11-34] | 22 [7-35] | 46 [29-60] | - |
| **10* | 23 [19-28] | ~45 [-] | 10 [3-18] | 65 [-] |  |  |  |  | 33 [15-56] | 33 [-] | 80^f^ [-] | 32 [11-34] | 37 [27-46] | 34 [17-49] | - |
| **17* | 29 [20-39] | - | 16 [6-25] | 39 [-] | - | - | - | - | - | 250 [-] | 420 [-] | 4 [-3-10] | 17 [3-30] | 1 [-14-13] | - |
| **29* | 29 [14-44] | - | 49 [28-70] | 58 [-] | - | - | - | - | - | 15 [-] | 10 [-] | 4 [-4-14] | - | 65 [40-103] | - |
| *35 | 68 [57-80] | ~75 [-] | - | 103 [-] | - | - | - | - | 57 [28-90] | - | - | - | - | - | - |
| **41* | 16 [13-19] | ~30 [-] | 11 [5-17] | 53 [-] | 9.5 [-] | 21 [14-29] | 26 [14-40] | 14 [1-29] | 16 [8-26] | - | 30 [-] | 9 [2-15] | 21 [10-31] | 21 [12-30] | 1.7 [-] |
| APA: Allelic percentage activity; CI: Confidence interval; LR: Linear regression; ML: Machine learning (neural network); PopPK: Population pharmacokinetic modelling; TAM: tamoxifen.  ^a^Nonfunctional alleles were not fixed to 0 but scaled to reference *CYP2D6*1* activity, thus no reference point is available for the lower end of the scale. This could lead to increased relative activities.  ^b^Study population likely overlapped with Jukić et al. ^c^Genotyping did not include *CYP2D6*2*. ^d^Two subvariants of *CYP2D6*2* were tested and different activities identified: Haplotypes *CYP2D6*2-rs5758550G* with activity reported above, and *CYP2D6*2-rs5758550A* with a percentage activity of 31% [17-49].  ^e^Some genotyping tests in multi-study analyses did not include *CYP2D6*2*.  ^f^Based on five or less patients. | | | | | | | | | | | | | | | |

**References**

1.	Mc Laughlin, A. M. *et al.* Nonlinear Mixed-Effects Model of Z-Endoxifen Concentrations in Tamoxifen-Treated Patients from the CEPAM Cohort. *Clin. Pharmacol. Ther.* **116**, 690–702 (2024).

2.	Puszkiel, A. *et al.* Factors Affecting Tamoxifen Metabolism in Patients With Breast Cancer: Preliminary Results of the French PHACS Study. *Clin. Pharmacol. Ther.* **106**, 585–595 (2019).

3.	Puszkiel, A. *et al.* Model-Based Quantification of Impact of Genetic Polymorphisms and Co-Medications on Pharmacokinetics of Tamoxifen and Six Metabolites in Breast Cancer. *Clin. Pharmacol. Ther.* **109**, 1244–1255 (2021).

4.	Antunes, M. V. *et al.* CYP3A4∗22 is related to increased plasma levels of 4-hydroxytamoxifen and partially compensates for reduced CYP2D6 activation of tamoxifen. *Pharmacogenomics* **16**, 601–617 (2015).

5.	Rae, J. M. *et al.* Cytochrome P450 2D6 activity predicts discontinuation of tamoxifen therapy in breast cancer patients. *Pharmacogenomics J.* **9**, 258–264 (2009).

6.	Areepium, N., Panomvana, D., Rungwanonchai, P., Sathaporn, S. & Voravud, N. Effects of CYP2D6 and UGT2B7 polymorphisms on pharmacokinetics of tamoxifen in Thai breast cancer patients. *Breast Cancer Targets Ther.* **5**, 73–78 (2013).

7.	Kiyotani, K. *et al.* Dose-adjustment study of tamoxifen based on CYP2D6 genotypes in Japanese breast cancer patients. *Breast Cancer Res. Treat.* **131**, 137–145 (2012).

8.	Arellano, C., Allal, B., Goubaa, A., Roché, H. & Chatelut, E. An UPLC-MS/MS method for separation and accurate quantification of tamoxifen and its metabolites isomers. *J. Pharm. Biomed. Anal.* **100**, 254–261 (2014).

9.	Antunes, M. V. *et al.* Ultra-high performance liquid chromatography tandem mass spectrometric method for the determination of tamoxifen, N-desmethyltamoxifen, 4-hydroxytamoxifen and endoxifen in dried blood spots - Development, validation and clinical application during breast. *Talanta* **132**, 775–784 (2015).

10.	Lee, K. H., Ward, B. A., Desta, Z., Flockhart, D. A. & Jones, D. R. Quantification of tamoxifen and three metabolites in plasma by high-performance liquid chromatography with fluorescence detection: Application to a clinical trial. *J. Chromatogr. B* **791**, 245–253 (2003).

11.	Zhu, Y. B., Zhang, Q., Zou, J. J., Yu, C. X. & Xiao, D. W. Optimizing high-performance liquid chromatography method with fluorescence detection for quantification of tamoxifen and two metabolites in human plasma: Application to a clinical study. *J. Pharm. Biomed. Anal.* **46**, 349–355 (2008).

12.	Borges, S. *et al.* Quantitative effect of CYP2D6 genotype and inhibitors on tamoxifen metabolism: Implication for optimization of breast cancer treatment. *Clin. Pharmacol. Ther.* **80**, 61–74 (2006).

13.	Barginear, M. F. *et al.* Increasing Tamoxifen Dose in Breast Cancer Patients Based on CYP2D6 Genotypes and Endoxifen Levels: Effect on Active Metabolite Isomers and the Antiestrogenic Activity Score. *Clin. Pharmacol. Ther.* **90**, 605–611 (2011).

14.	Irvin, W. J. *et al.* Genotype-guided tamoxifen dosing increases active metabolite exposure in women with reduced CYP2D6 metabolism: A multicenter study. *J. Clin. Oncol.* **29**, 3232–3239 (2011).

15.	Marcath, L. A. *et al.* Comprehensive assessment of cytochromes P450 and transporter genetics with endoxifen concentration during tamoxifen treatment. *Pharmacogenet. Genomics* **27**, 402–409 (2017).

16.	Fox, P. *et al.* Dose escalation of tamoxifen in patients with low endoxifen level: Evidence for therapeutic drug monitoring - The TADE study. *Clin. Cancer Res.* **22**, 3164–3171 (2016).

17.	Fernández-Santander, A. *et al.* Relationship between Genotypes Sult1a2 and Cyp2d6 and Tamoxifen Metabolism in Breast Cancer Patients. *PLoS ONE* **8**, e70183 (2013).

18.	Jaremko, M., Kasai, Y., Barginear, M. F., Raptis, G., Desnick, R. J. & Yu, C. Tamoxifen metabolite isomer separation and quantification by liquid chromatography-tandem mass spectrometry. *Anal. Chem.* **82**, 10186–10193 (2010).

19.	Hertz, D. L. *et al.* *In vivo* assessment of the metabolic activity of CYP2D6 diplotypes and alleles. *Br. J. Clin. Pharmacol.* **80**, 1122–1130 (2015).

20.	Teunissen, S. F. *et al.* Development and validation of a quantitative assay for the analysis of tamoxifen with its four main metabolites and the flavonoids daidzein, genistein and glycitein in human serum using liquid chromatography coupled with tandem mass spectrometry. *J. Chromatogr. B* **877**, 2519–2529 (2009).

21.	Woo, H. I. *et al.* Variations in plasma concentrations of tamoxifen metabolites and the effects of genetic polymorphisms on tamoxifen metabolism in Korean patients with breast cancer. *Oncotarget* **8**, 100296–100311 (2017).

22.	Love, R. R. *et al.* CYP2D6 genotypes, endoxifen levels, and disease recurrence in 224 Filipino and Vietnamese women receiving adjuvant tamoxifen for operable breast cancer. *SpringerPlus* **2**, 52 (2013).

23.	Hennig, E. E. *et al.* Limited predictive value of achieving beneficial plasma (Z)-endoxifen threshold level by CYP2D6 genotyping in tamoxifen-treated Polish women with breast cancer. *BMC Cancer* **15**, 570 (2015).

24.	Powers, J. L. *et al.* Multigene and Drug Interaction Approach for Tamoxifen Metabolite Patterns Reveals Possible Involvement of CYP2C9, CYP2C19, and ABCB1. *J. Clin. Pharmacol.* **56**, 1570–1581 (2016).

25.	Helland, T. *et al.* Serum concentrations of active tamoxifen metabolites predict long-term survival in adjuvantly treated breast cancer patients. *Breast Cancer Res.* **19**, 125 (2017).

26.	Gjerde, J. *et al.* Effects of CYP2D6 and SULT1A1 genotypes including SULT1A1 gene copy number on tamoxifen metabolism. *Ann. Oncol.* **19**, 56–61 (2008).

27.	Lim, H. S., Lee, H. J., Lee, K. S., Lee, E. S., Jang, I. J. & Ro, J. Clinical implications of CYP2D6 genotypes predictive of tamoxifen pharmacokinetics in metastatic breast cancer. *J. Clin. Oncol.* **25**, 3837–3845 (2007).

28.	Mürdter, T. E. *et al.* Activity Levels of Tamoxifen Metabolites at the Estrogen Receptor and the Impact of Genetic Polymorphisms of Phase I and II Enzymes on Their Concentration Levels in Plasma. *Clin. Pharmacol. Ther.* **89**, 708–717 (2011).

29.	Saladores, P. *et al.* Tamoxifen metabolism predicts drug concentrations and outcome in premenopausal patients with early breast cancer. *Pharmacogenomics J.* **15**, 84–94 (2015).

30.	Sanchez-Spitman, A. *et al.* Tamoxifen Pharmacogenetics and Metabolism: Results From the Prospective CYPTAM Study. *J. Clin. Oncol.* **37**, 636–646 (2019).

31.	Gjerde, J., Kisanga, E. R., Hauglid, M., Holm, P. I., Mellgren, G. & Lien, E. A. Identification and quantification of tamoxifen and four metabolites in serum by liquid chromatography-tandem mass spectrometry. *J. Chromatogr. A* **1082**, 6–14 (2005).

32.	Ximenez, J. P. B., De Andrade, J. M., Marques, M. P., Coelho, E. B., Suarez-Kurtz, G. & Lanchote, V. L. Hormonal status affects plasma exposure of tamoxifen and its main metabolites in tamoxifen-treated breast cancer patients. *BMC Pharmacol. Toxicol.* **20**, 81 (2019).

33.	Safgren, S. L. *et al.* Evaluation of CYP2D6 enzyme activity using a dextromethorphan breath test in women receiving adjuvant tamoxifen. *Pharmacogenet. Genomics* **25**, 157–163 (2015).

34.	Helland, T. *et al.* Drug monitoring of tamoxifen metabolites predicts vaginal dryness and verifies a low discontinuation rate from the Norwegian Prescription Database. *Breast Cancer Res. Treat.* **177**, 185–195 (2019).

35.	Madlensky, L. *et al.* Tamoxifen Metabolite Concentrations, *CYP2D6* Genotype, and Breast Cancer Outcomes. *Clin. Pharmacol. Ther.* **89**, 718–725 (2011).

36.	DeCensi, A. *et al.* Association of CYP2D6 genotype and tamoxifen metabolites with breast cancer recurrence in a low-dose trial. *Npj Breast Cancer* **7**, 34 (2021).

37.	Thorén, L. *et al.* Impairment of endoxifen formation in tamoxifen-treated premenopausal breast cancer patients carrying reduced-function CYP2D6 alleles. *Br. J. Clin. Pharmacol.* **87**, 1243–1252 (2021).

38.	Decensi, A. *et al.* Randomized double-blind 2 X 2 trial of low-dose tamoxifen and fenretinide for breast cancer prevention in high-risk premenopausal women. *J. Clin. Oncol.* **27**, 3749–3756 (2009).

39.	Jorge-Aarón, R. M., Rodrigo, R. C. & Esther, M. P. R. Endoxifen levels and metabolic phenotype-associated factors in Mexican Mestizo patients under tamoxifen treatment. *Pharmacogenomics* **21**, 929–943 (2020).

40.	Hussaarts, K. G. A. M. *et al.* Impact of curcumin (With or without piperine) on the pharmacokinetics of tamoxifen. *Cancers* **11**, 403 (2019).

41.	Braal, C. L. *et al.* Influence of green tea consumption on endoxifen steady-state concentration in breast cancer patients treated with tamoxifen. *Breast Cancer Res. Treat.* **184**, 107–113 (2020).

42.	Rangel-Méndez, J. A., Graniel-Sabido, M. J., Sánchez-Cruz, J. F. & Moo-Puc, R. Development of a high-performance liquid chromatography method with fluorescence detection for the routine quantification of tamoxifen, endoxifen and 4-hydroxytamoxifen in plasma from breast cancer patients. *Biomed. Chromatogr.* **33**, e4462 (2019).

43.	Binkhorst, L. *et al.* Quantification of tamoxifen and three of its phase-I metabolites in human plasma by liquid chromatography/triple-quadrupole mass spectrometry. *J. Pharm. Biomed. Anal.* **56**, 1016–1023 (2011).

44.	Buck, S. A. J. *et al.* Influence of probenecid on endoxifen systemic exposure in breast cancer patients on adjuvant tamoxifen treatment. *Ther. Adv. Med. Oncol.* **14**, 17588359221081075 (2022).

45.	Buijs, S. M. *et al.* CBD-oil as a potential solution in case of severe tamoxifen-related side effects. *Npj Breast Cancer* **9**, 63 (2023).

46.	Braal, C. L. *et al.* Therapeutic Drug Monitoring of Endoxifen for Tamoxifen Precision Dosing: Feasible in Patients with Hormone-Sensitive Breast Cancer. *Clin. Pharmacokinet.* **61**, 527–537 (2022).

47.	Braal, C. L. *et al.* Factors affecting inter-individual variability in endoxifen concentrations in patients with breast cancer: results from the prospective TOTAM trial. *Breast Cancer Res. Treat.* **195**, 65–74 (2022).

48.	Buijs, S. M. *et al.* The impact of endoxifen-guided tamoxifen dose reductions on endocrine side-effects in patients with primary breast cancer. *ESMO Open* **8**, 100786 (2023).

49.	Almeida, T. *et al.* (Z)-Endoxifen and Early Recurrence of Breast Cancer: An Explorative Analysis in a Prospective Brazilian Study. *J. Pers. Med.* **12**, 511 (2022).

50.	Chiwambutsa, S. M. *et al.* Effects of Genetic Polymorphisms of Drug Metabolizing Enzymes and co-Medications on Tamoxifen Metabolism in Black South African Women with Breast Cancer. *Clin. Pharmacol. Ther.* **114**, 127–136 (2023).

51.	Kiyotani, K. *et al.* Significant effect of polymorphisms in CYP2D6 and ABCC2 on clinical outcomes of adjuvant tamoxifen therapy for breast cancer patients. *J. Clin. Oncol.* **28**, 1287–1293 (2010).

52.	Scott, S. A., Edelmann, L., Kornreich, R., Erazo, M. & Desnick, R. J. *CYP2C9*, *CYP2C19* and *CYP2D6* Allele Frequencies in the Ashkenazi Jewish Population. *Pharmacogenomics* **8**, 721–730 (2007).

53.	Chen, Y. *et al.* Effect of Genetic Variability in 20 Pharmacogenes on Concentrations of Tamoxifen and Its Metabolites. *J. Pers. Med.* **11**, 507 (2021).

54.	Johansson, H. *et al.* A pooled analysis of CYP2D6 genotype in breast cancer prevention trials of low-dose tamoxifen. *Breast Cancer Res. Treat.* **159**, 97–108 (2016).

55.	Nardin, J. M. *et al.* The Influences of Adherence to Tamoxifen and CYP2D6 Pharmacogenetics on Plasma Concentrations of the Active Metabolite (Z)-Endoxifen in Breast Cancer. *Clin. Transl. Sci.* **13**, 284–292 (2020).

56.	Chen, S. *et al.* A genomic mutational constraint map using variation in 76,156 human genomes. *Nature* **625**, 92–100 (2024).

57.	gnomAD. <https://gnomad.broadinstitute.org/> (2024). Accessed 5 December 2025.

58.	Van der Lee, M. *et al.* Toward predicting CYP2D6-mediated variable drug response from CYP2D6 gene sequencing data. *Sci. Transl. Med.* **13**, eabf3637 (2021).

59.	Agema, B. C. *et al.* Toward model-informed precision dosing for tamoxifen: A population-pharmacokinetic model with a continuous CYP2D6 activity scale. *Biomed. Pharmacother.* **160**, 114369 (2023).

60.	Haslemo, T., Eliasson, E., Jukić, M. M., Ingelman-Sundberg, M. & Molden, E. Significantly lower CYP2D6 metabolism measured as the O/N-desmethylvenlafaxine metabolic ratio in carriers of *CYP2D6*41* versus *CYP2D6*9* or *CYP2D6*10*: a study on therapeutic drug monitoring data from 1003 genotyped Scandinavian patients. *Br. J. Clin. Pharmacol.* **85**, 194–201 (2019).

61.	Jukić, M. M., Smith, R. L., Molden, E. & Ingelman-Sundberg, M. Evaluation of the *CYP2D6* Haplotype Activity Scores Based on Metabolic Ratios of 4,700 Patients Treated With Three Different CYP2D6 Substrates. *Clin. Pharmacol. Ther.* **110**, 750–758 (2021).

62.	Størset, E., Bråten, L. S., Ingelman-Sundberg, M., Johansson, I., Molden, E. & Kringen, M. K. Impact of *CYP2D6*2*, *CYP2D6*35*, rs5758550, and related haplotypes on risperidone clearance in vivo. *Eur. J. Clin. Pharmacol.* **80**, 1531–1541 (2024).

63.	Kehinde, O. *et al.* Cytochrome P450 2D6 *17 and *29 Allele Activity for Risperidone Metabolism: Advancing Precision Medicine Health Equity. *Clin. Pharmacol. Ther.* **118**, 1152–1160 (2025).

64.	Bennett, M. H. *et al.* Impact of *CYP2D6* Genotype and Inhibitor Use on Risperidone Metabolism in Children: Functional Insights Into the **17* and **29* Alleles. *Clin. Transl. Sci.* **19**, e70525 (2026).

65.	Frederiksen, T., Areberg, J., Raoufinia, A., Schmidt, E., Stage, T. B. & Brøsen, K. Estimating the *In Vivo* Function of *CYP2D6* Alleles through Population Pharmacokinetic Modelling of Brexpiprazole. *Clin. Pharmacol. Ther.* **113**, 360–369 (2023).

66.	Frederiksen, T., Areberg, J., Schmidt, E., Bjerregaard Stage, T. & Brøsen, K. Quantification of *In Vivo* Metabolic Activity of *CYP2D6* Genotypes and Alleles Through Population Pharmacokinetic Analysis of Vortioxetine. *Clin. Pharmacol. Ther.* **109**, 150–159 (2021).

67.	Frederiksen, T., Areberg, J., Schmidt, E., Stage, T. B. & Brøsen, K. Cytochrome P450 *2D6* genotype-phenotype characterization through population pharmacokinetic modeling of tedatioxetine. *CPT Pharmacomet. Syst. Pharmacol.* **10**, 983–993 (2021).

68.	Abduljalil, K. *et al.* Assessment of Activity Levels for *CYP2D6*1*, *CYP2D6*2*, and *CYP2D6*41* Genes by Population Pharmacokinetics of Dextromethorphan. *Clin. Pharmacol. Ther.* **88**, 643–651 (2010).
